# Lignocellulose-Based Optical Biofilter with High Near-Infrared Transmittance via Lignin Capturing–Fusing Approach

**DOI:** 10.34133/research.0250

**Published:** 2023-10-16

**Authors:** Shixu Yu, Yifang Zhou, Meixue Gan, Lu Chen, Yimin Xie, Yuning Zhong, Qinghua Feng, Chaoji Chen

**Affiliations:** ^1^ Hubei Provincial Key Laboratory of Green Materials for Light Industry, Hubei University of Technology, Wuhan 430068, China.; ^2^ Hubei Biomass-Resource Chemistry and Environmental Biotechnology Key Laboratory, School of Resource and Environmental Sciences, Wuhan University, Wuhan 430079, China.; ^3^ Hubei Open University, Wuhan 430074, China.

## Abstract

Near-infrared (NIR) transparent optical filters show great promise in night vision and receiving windows. However, NIR optical filters are generally prepared by laborious, environmentally unfriendly processes that involve metal oxides or petroleum-based polymers. We propose a lignin capturing–fusing approach to manufacturing optical biofilters based on molecular collaboration between lignin and cellulose from waste agricultural biomass. In this process, lignin is captured via self-assembly in a cellulose network; then, the lignin is fused to fill gaps and hold the cellulose fibers tightly. The resulting optical biofilter featured a dense structure and smooth surface with NIR transmittance of ~90%, ultralow haze of close to 0%, strong ultraviolet-visible light blocking (~100% at 400 nm and 57.58% to 98.59% at 550 nm). Further, the optical biofilter has comprehensive stability, including water stability, solvent stability, thermal stability, and environmental stability. Because of its unique properties, the optical biofilter demonstrates potential applications in the NIR region, such as an NIR-transmitting window, NIR night vision, and privacy protection. These applications represent a promising route to produce NIR transparent optical filters starting from lignocellulose biomass waste.

## Introduction

Near-infrared (NIR) transparent optical filters with low haze and dark color have attracted interest in applications such as night vision, privacy protection, and medicolegal identification [1,2]. These filters are composed of metal oxides, germanium/chalcogenide glasses, or silicon semiconductors that are expensive, environmentally harmful, and/or complicated to fabricate [3]. To circumvent these shortcomings, researchers have embedded dyes that absorb ultraviolet-visible (UV-vis) light and allow passage of NIR light into polymer matrices or inorganic/organic hybrid polymers [1,2,4,5]. Despite improved optical performance, the incorporation of dyes poses problems with safety, sustainability, and NIR transmittance mainly attributed to toxicity of dyes and poor balance between UV-vis and NIR transmittance.

Lignocellulose-based biosourced materials are abundant, renewable, nontoxic, and mechanically strong candidates for optical materials [6–13]. However, as an inexhaustible resource [14–17], lignocellulose from agricultural bioresource is wasted in large quantities [18]. In addition, optically, lignocellulose is mainly involved in UV [19–21] or visible regions [22–24] according to the presence or absence of lignin [25]. Because many types of extracted lignin are dark in color [26], lignin can theoretically function as a natural dye in cellulose films to produce lignin–cellulose composite films that have low haze and dark color, which are requirements of NIR transparent optical filters. However, lignin in cellulose aggregates, thereby compromising interior compactness and then producing optical haze [27]. In addition, surface smoothness is a key factor that must be considered in reducing the haze of lignocellulose-based NIR transparent materials [28]. Therefore, effective engineering strategies are needed to tune the structure of lignin–cellulose composite films, especially the synergistic interaction between the lignin and cellulose components, for NIR transparent optical filter applications.

**Fig. 1.**
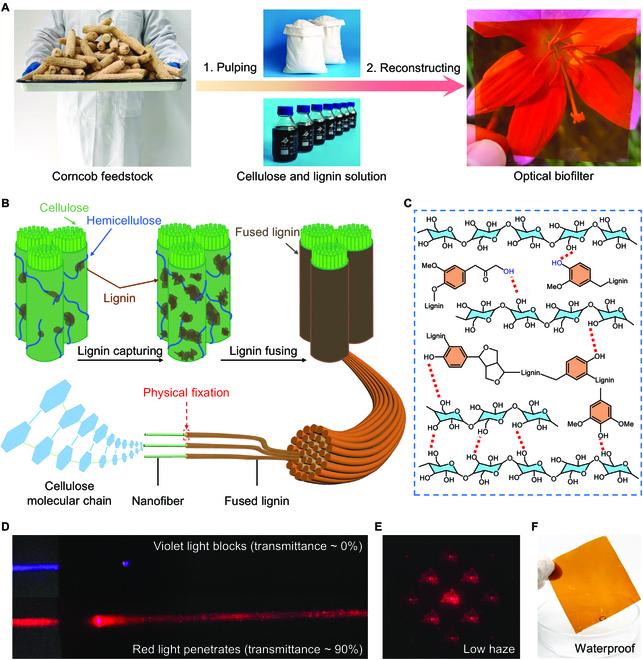
Separation and recombination technology for optical biofilter. (A) The process from waste corncob to optical biofilter. (B) Microstructure diagram of lignin capturing–fusing approach for optical biofilter fabrication. Initially, lignin occupies the pores of a cellulose network via solvent/antisolvent self-assembly. Then a lignin–cellulose film is obtained after preliminary drying. In step 2, hot pressing promotes the fusion and uniform distribution of lignin, resulting in a homogeneous and dense structure of optical biofilter. (C) The interaction between fused lignin and cellulose. ^1^H–^13^C NMR revealed the emergence of new hydroxyl groups (blue) that created more hydrogen bonds between lignin and cellulose, further strengthening their interaction. (D to F) The main properties of optical biofilter, including selective transmittance of light (D), low haze (E), and water immunity (F).

To address these essential needs, we propose a facile lignin capturing–fusing strategy to construct an NIR transparent lignocellulose-based optical biofilter directly from waste corncob (Fig. 1A and B). The biofilter achieved selective light transmission, i.e., high NIR transmittance, UV-vis light shielding, and ultralow optical haze. The design of this lignin capturing–fusing approach first profits from the solvent/antisolvent self-assembling ability of lignin that originates from its amphiphilicity, e.g., tetrahydrofuran/water and acetic acid/water self-assembly (Fig. S1) [29,30]. In this manner, a cellulose fiber network can easily capture assembled lignin colloid spheres (Fig. S2) that enables the lignin to spontaneously occupy vacant space in the cellulose network. In addition, disrupted hydrogen bonding and increased free volume caused by acetylation prompt the captured lignin to be fused to fill gaps during hot pressing (a property unique to acetic acid lignin) [31,32], which then holds the fibers tightly via abundant hydrogen bonds (Fig. 1C), resulting in a homogeneous dense structure (Figs. S3 to S5). Therefore, the glassy optical biofilters (Fig. S6) have high NIR transmittance, selective light transmission, ultralow haze, and outstanding water resistance (Fig. 1D to F). With all these advantages, the optical biofilter shows the potential to be a promising candidate for optical materials used in the NIR region. In addition, our lignin capturing–fusing approach promotes use of waste agricultural biomass, opening the way to develop sustainable NIR optical materials.

## Results

### Construction of optical biofilter from waste corncob

In our lignin capturing–fusing approach, a key requirement is enabling a viscous flow state of the lignin in the cellulose network. Therefore, we used acetic acid pulping to process waste corncob into cellulose and partially acetylated lignin (Notes S1 and S2). Compared with nonacetylated lignin, the acetic acid lignin became viscous at 180 °C (Fig. S7). Typically, lignin capturing and fusion were achieved by lignin self-assembly and hot pressing (180 °C, 5 MPa), respectively. Cellulose hydrogels were immersed in lignin/acetic acid solution to exchange liquid. Then, lignin–cellulose hydrogels were obtained after reimmersing the gels in deionized water to remove excess acetic acid, followed by a preliminary drying process to obtain lignin–cellulose films. Finally, lignocellulose-based optical biofilters were fabricated via hot pressing. The resulting dense structure and smooth surface enabled desirable transparency of the biofilter (Fig. S8) with high NIR transmittance (~90%) and ultralow haze (~0%), fulfilling the prerequisites for NIR transparent material.

### Structure and molecular interaction of optical biofilter

**Fig. 2.**
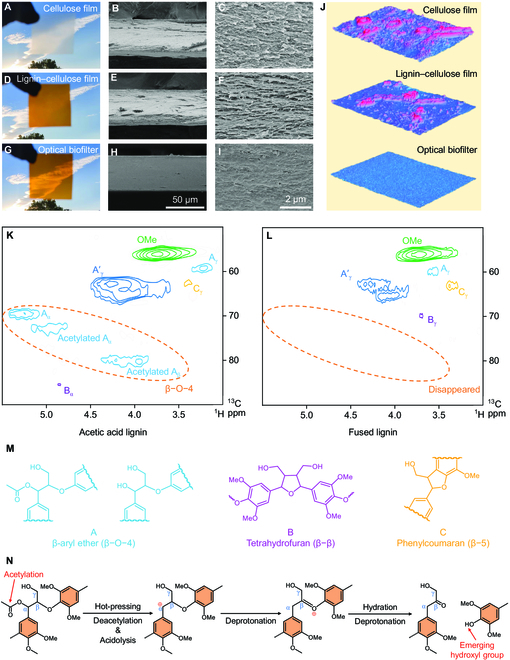
Structure of the optical biofilter and fused lignin. (A to I) Digital and scanning electron microscopy images of cellulose film (A to C), lignin–cellulose film (D to F), and optical biofilter (G to I). (J) Raman surface 2D imaging of cellulose film, lignin–cellulose film, and optical biofilter according to difference of height. Red indicates a higher position; blue indicates a lower position. (K and L) 2D-HSQC NMR spectra of acetic acid lignin (K) and the fused lignin (L) in aliphatic regions (δ_C_/δ_H_ of 50 to 90/3.0 to 5.5). (M) Structures of lignin bonds. (N) The mechanism of cleaving the lignin β−O−4 bonds and regenerating hydroxyl groups during the lignin fusing process. We chose the alpha-site acetylated β−O−4 structure to demonstrate the cleavage of β−O−4 bonds and the formation of new hydroxyl groups.

The transparent optical biofilter featured a more homogeneous and denser structure with smoother surface compared with cellulose film and lignin–cellulose film (Fig. 2A to J and Fig. S9). These improvements were attributed to the self-assembled, fused lignin that acted as a binder and bonded with cellulose [33]. Without lignin, hot-pressed cellulose films had a loose laminated structure (Fig. S10A to F), which indicated the necessity of lignin capturing for the dense structure of the film. In addition, to investigate the contribution of lignin fusion, we hot-pressed the lignin–cellulose film at 130 °C, a temperature higher than the glass transition temperature (*T*_g_) and lower than the viscous flow temperature (*T*_f_) of lignin (Fig. S7). The film had enhanced compactness but defects remained (Fig. S10G to I). Thus, both capturing and fusing of lignin are keys to the homogeneous dense structure of the optical biofilter. Note that our lignin capturing–fusing approach is friendly to the structural integrity of the cellulose matrix as verified by the similar x-ray diffraction patterns of cellulose film, lignin–cellulose film, and optical biofilter (Fig. S11).

To investigate the chemical evolution of the optical biofilter during the pulping and reconstruction, we conducted Fourier transform infrared (FTIR) spectroscopy and ^1^H–^13^C nuclear magnetic resonance (NMR). FTIR spectroscopy (Fig. S12) showed that the lignin–cellulose film and optical biofilter each had a new peak at 1,510 cm^−1^ from the vibrations of the aromatic rings of captured lignin [34]. In addition, another new absorption peak in lignin, lignin–cellulose film, and optical biofilter appeared at 1,710 cm^−1^, which confirmed the partial acetylation of acetic acid lignin [35]. The NMR spectra in the aliphatic region (δ_C_/δ_H_ of 50 to 90/3.0 to 5.5) decisively confirmed the transformation of lignin and fused lignin and the interaction between fused lignin and cellulose (Fig. 2K to M). After lignin fusion, the signals correlating to A_α_ (δ_C_/δ_H_ of 69.3/5.23), acetylated A_α_ (δ_C_/δ_H_ of 72.6/4.86), and acetylated A_β_ (δ_C_/δ_H_ of 80.1/3.95) disappeared in the fused lignin, which demonstrated the cleavage of the acetyl groups and β−O−4 ether bonds. The lignin acetyl groups were detached because of the harsh pressure and temperature. This detachment led to acidolysis of lignin that eventually produced ketones and phenols, resulting in newly available hydroxyl groups that strengthened hydrogen bonding between the lignin and cellulose in the optical biofilter (Figs. 1C and 2N) [34,36,37].

### Optical properties of the optical biofilter

**Fig. 3.**
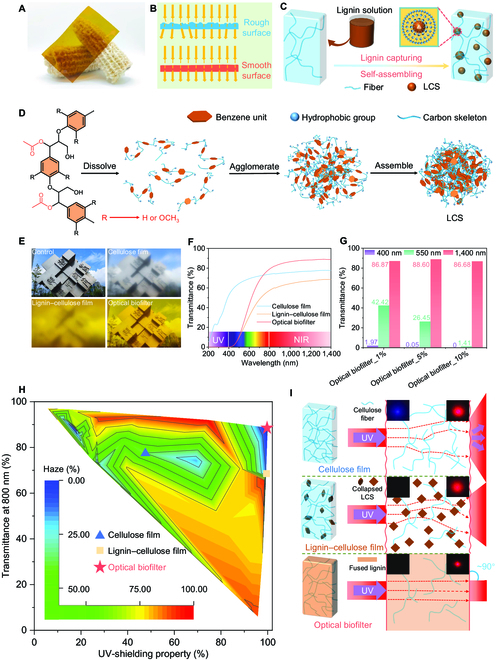
Optical properties of biofilter. (A) Digital image of optical biofilter with excellent transparency. (B) Schematic diagram of how light is affected by surface morphology during propagation. (C and D) Mechanism of lignin capturing based on self-assembly in cellulose hydrogel. (E) Digital images with camera lens covered by cellulose film, lignin–cellulose film, and optical biofilter. (F) Transmittance of films from 200 to 1,400 nm. (G) Transmittance of optical biofilter_1%, optical biofilter_5%, and optical biofilter_10% at 400, 550, and 1,400 nm. (H) Transmittance at 800 nm, UV shielding, and haze at 550-nm properties of biofilter and other lignocellulose-based materials. (I) Schematic of the effects of components and structure on light propagation at different wavelengths. LCS, lignin colloid sphere.

Smooth surface and dense interior are key factors to realize the transparency of the optical biofilter. A smooth surface enabled the biofilter to reduce light reversal because of a uniform surface height that was attributed to the surface rearrangement during lignin fusion (Fig. 3A and B). In addition, the innovative use of lignin solvent/antisolvent self-assembly inside the cellulose hydrogel (Fig. 3C and D) enabled the lignin to transform into lignin colloid spheres that then occupied the pores of cellulose. This occupation initially achieved a uniform distribution of lignin that was crucial for the production of a dense structure. As a result, compared with cellulose film and lignin–cellulose film, the optical biofilter enabled one to see the view behind without fuzziness (Fig. 3E).

To further evaluate the unique optical properties of the biofilter, we measured the transmittance (200 to 1,400 nm) and haze (550 nm) of the films. UV-vis-NIR spectrophotometry demonstrated that lignin capturing process was the major contributor to the UV-blocking property of both lignin–cellulose film and optical biofilter, enabling resistance of almost all UV light (>99.9%; Fig. 3F and Fig. S13) [38], thus preventing UV-responsive paint from color changing (Fig. S14). After the lignin capturing process, the visible transmittance decreased from 67.25% to 20.46% at 550 nm and the NIR transmittance decreased from 77.58% to 68.40% at 1,400 nm, respectively. By contrast, the optical biofilter had equally strong UV-shielding ability with a dramatically enhanced NIR transmittance of 88.60% that was attributed to lignin fusion and that was unaffected by the concentration of the aqueous lignin/acetic acid solution (Fig. 3G, Figs. S15 and S16, and Note S3). These results also indicated that the optical blocking properties in the visible region could be tailored by adjusting the lignin loading concentration. When the concentration of the lignin solution came to 10 wt%, the optical biofilter resisted most of visible light (98.59% at 550 nm), featuring simultaneous UV and visible lights blocking. The optical biofilter also featured ultralow haze of ~0% at 550 nm, which was substantially lower than that of the cellulose film (~40%) and the lignin–cellulose film (~15%) (Figs. S17 and S18). More interestingly, unlike other lignocellulose-based materials (for example, transparent wood [39,40] and lignin-incorporated cellulose film [27,41]), our biofilter simultaneously exhibited high NIR transmittance, ultralow haze, and strong UV resistance (Fig. 3H and Table S1).

Figure 3I schematically shows that the effect of lignin capturing and fusing processes on the unique optical properties of the biofilter. In the capturing process, lignin embedded in cellulose network ensured the lignin–cellulose film to absorb UV-vis light; however, lignin did not efficiently reduce light scattering. After lignin fusion, UV-blocking property of optical biofilter endowed by lignin capturing process was not compromised; meanwhile, the lignin held fibers tightly together, forming a uniform and dense structure with smooth surface that allowed light to avoid passing through air and solids as frequently as in the cellulose and lignin–cellulose films. Thus, no massive light scattering occurred. In addition, the optical biofilters fabricated on the basis of the lignin capturing–fusing approach but using bamboo and wood exhibited transparency similar to the corncob-based biofilter, which indicated the generality of our strategy (Figs. S19 to S23 and Notes S4 to S7).

### Comprehensive stability of the optical biofilter

**Fig. 4.**
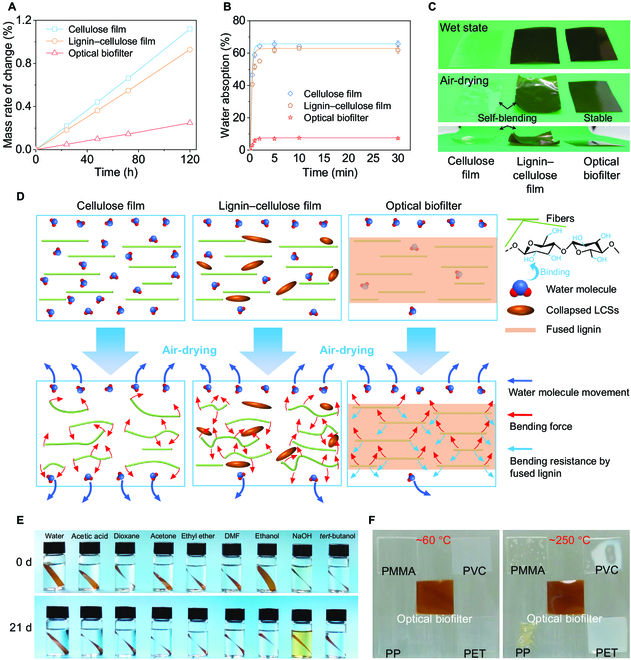
Comprehensive stability of the optical biofilter. (A) Water uptake of CaCl_2_ in bottles cover by cellulose film, lignin–cellulose film, and optical biofilter during 120 h. (B) Water absorption after immersing in deionized water for 30 min. (C) Digital images of cellulose film, lignin–cellulose film, and optical biofilter at drying state after immersing in deionized water. (D) Mechanism of antiwater property of biofilter. (E) Solvent stability in the order of deionized water, 90 vol% of aqueous acetic acid, dioxane, acetone, ethyl ether, *N*,*N*-dimethylformamide (DMF), ethanol, 1 wt% of aqueous NaOH , tertiary butanol (*tert*-butanol). (F) Thermal stability of optical biofilter compared with polymethyl methacrylate (PMMA), polyvinyl chloride (PVC), polypropylene (PP), and polyethylene glycol terephthalate (PET).

Because structural stability is crucial for practical applications, we measured water stability, solvent stability, thermal stability, and environmental stability of the lignocellulose-based optical biofilter. Compared with cellulose and lignin–cellulose films, the biofilter exhibited a reduced water vapor transmission rate attributed to its homogeneous dense structure (Fig. 4A and Figs. S24 to S26). In addition, the optical biofilter had low water absorption of ~7.45%, considerably lower than that of cellulose film (~65.78%), lignin–cellulose film (~61.86%), and other lignocellulose-based materials, such as hot-pressed films [42,43], superhydrophobic coating materials [44], and lignin–cellulose composite bioplastics [34] (Fig. 4B and Fig. S27). After the water absorption test, we dried the films. The dried optical biofilter did not exhibit any obvious shape change, which demonstrated the excellent dimensional stability (Fig. 4C) and the feasibility of direct use in humid environments. Figure 4D shows the mechanism of water stability of the biofilter. The dense structure of the biofilter prevented it from water permeation, further decreasing the water vapor transmission rate and water absorption. The fused lignin generated bending resistance that limited the rearrangement of the cellulose fibers, resulting in an unchanged shape. In addition, the water contact angle of the lignin–cellulose film and optical biofilter increased with the presence of lignin (Fig. S28).

To further investigate stability, we soaked the optical biofilter for 21 d in deionized water, aqueous acetic acid (the solution used to dissolve lignin in this work), dioxane, acetone, ethyl ether, *N*,*N*-dimethylformamide, ethanol, aqueous 1 wt% of NaOH, and tertiary butanol (Fig. 4E). The biofilter had excellent stability in all the solvents except that lignin partially dissolved in the NaOH solution. Moreover, lignin, which is more thermoduric than cellulose [42], provided the optical biofilter with a higher thermal weight loss temperature (Fig. S29). Compared with common plastics, such as polymethyl methacrylate, polyvinyl chloride, polypropylene, and polyethylene glycol terephthalate, the optical biofilter did not exhibit any visible change between 60 and 250 °C (Fig. 4F). We also exposed the biofilter to a soil environment with sunshine and simulated rainfall for 150 d; no visible alteration appeared on the biofilter surface (Fig. S30), which demonstrated excellent environmental stability indicative of the antioxidant, antibacterial, UV-resistant, and waterproof properties of lignin. Note that the lignin capturing–fusing approach indeed caused decreased mechanical strength (~78 MPa) because of the reduction of hydrogen bonds between cellulose chains. However, the resulting mechanical strength was still greater than that of common petroleum-based plastics. (Figs. S31 and S32). Meanwhile, the biofilter also demonstrated good recyclability (Fig. S33). Through a recycling step of cellulose, the remanufactured cellulose hydrogel could be reapplied to construct biofilter via our lignin capturing–fusing approach.

### Applications of optical biofilter

**Fig. 5.**
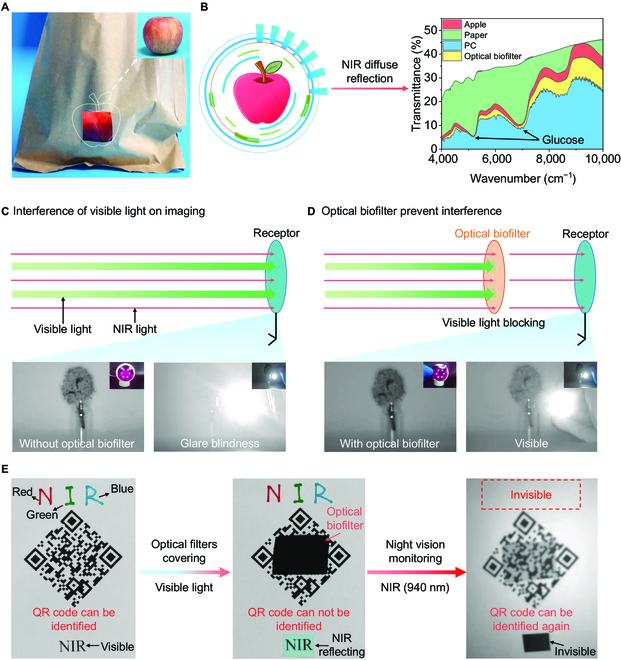
Applications of the optical biofilter in NIR region. (A and B) Optical biofilter_5% used in the NIR transmitting window for fruit detection. (C and D) Optical biofilter_10% used in prevention of glare with NIR night vision monitor. (E) Optical biofilter_10% used in masking sensitive information.

Given the excellent properties that met the demands of practical NIR transparent materials, we tested applications of the optical biofilter. To prevent blue light damage to the eyes, we fashioned the biofilter into eyeglass lenses (Fig. S34). In addition, the biofilter functioned as a window for NIR diffuse spectroscopy to determine the diffuse reflection of an apple (Fig. 5A). Compared with the original paper fruit sleeve and commercial polycarbonate NIR transparent film, the optical biofilter exhibited the preponderance of accurate detection due to the highly consistent diffuse reflectance spectra with apple (Fig. 5B). We also noted that an NIR night vision monitor was blinded by glare from strong light exposure (Fig. 5C). After covering with the optical biofilter, the monitor maintained normal operation (Fig. 5D and Fig. S35). In addition, the optical biofilter provided privacy security (Fig. 5E). In visible light, the biofilter prevented a quick response (QR) code from being recognized, which indicated its effectiveness in information protection. After switching to a night vision model, the words with 3 different colors (red, green, and blue) and the words covered by the NIR-reflecting filter became invisible. However, the QR code reappeared and the words “Privacy Information” stored in code can be identified by a recognizer, which indicated the effectiveness of determination of privacy information in NIR light.

## Discussion

We demonstrated a novel lignin capturing–fusing approach to manufacturing an optical biofilter for use as NIR transparent material. The captured lignin was fused to fill the gaps in a cellulose network, which then held the fibers tightly and created a homogeneous dense structure. Both the lignin and the dense structure provided the biofilter with unique optical properties, including strong UV-vis light blocking (~100% at 400 nm and 57.58% to 98.59% at 550 nm), low haze (close to 0%), and high NIR transmittance (~90%). In addition, the biofilter had excellent, comprehensive stability, including water stability, solvent stability, thermal stability, and environmental stability. Thus, the multifunctional optical biofilter had many applications in optics, especially in the NIR region, such as reducing blue light, forming an NIR transmitting window for fruit detection, preventing an NIR night vision monitor from glare blindness, and protecting sensitive information. This proposed lignin capturing–fusing approach for high-performance optical biofilter fabrication demonstrates a pathbreaking opportunity for lignocellulose-based biomass to broaden applications in optics.

## Materials and Methods

### Materials

Benzyl-trimethyl ammonium hydroxide (BzMe_3_NOH; 40 wt% of aqueous solution) was purchased from TCI Shanghai Co. Ltd. (Shanghai, China). Ethylene glycol (98 wt%), *tert*-butanol (≥99.5%), and anhydrous calcium chloride (CaCl_2_; 96%) were supplied by Shanghai Macklin Biochemical Co. Ltd. (Shanghai, China). Glacial acetic acid, sodium acetate, sulfuric acid, hydrochloric acid, dioxane, acetone, ethyl ether, *N*,*N*-dimethylformamide, ethanol, sodium hydroxide, and urea were purchased from Sinopharm Chemical Reagent Co. Ltd. (Shanghai, China). Cellulose and lignin were extracted from corncob waste (Notes S1 and S2).

### Preparation of optical biofilter

The optical biofilter was obtained by a facile strategy without any chemical complexities. Typically, cellulose (1.8 g) extracted from corncob was swelled in deionized water (7.5 g) and dissolved with 40 wt% of aqueous BzMe_3_NOH (22.5 g) at 8 °C. The mixture was further incubated at 8 °C for 24 h, centrifuged at 6,000 rpm for 5 min, cast with a height of 1 mm, and regenerated in ethylene glycol at 8 °C. The resulting cellulose hydrogel was washed with deionized water to remove chemicals. Then, the hydrogel was immersed in aqueous 5 wt% of lignin/acetic acid (90 vol%) for 5 h (hydrogel: lignin solution ≈ 1 g: 20 ml) to exchange the solution, followed by immersion in deionized water to remove acetic acid (lignin capturing process). The subsequent lignin–cellulose hydrogel was dried at 97 °C using a rapid Köthen sheet former (RK-3A, FrankPTI, Vorchdorf, Austria) to manufacture a lignin–cellulose film. Finally, the lignin–cellulose film was hot-pressed between 2 polyimide films at 180 °C and 5 MPa for 1 h to produce optical biofilter (lignin fusing process). Biofilters with different lignin loading contents were produced by using acetic acid solutions with lignin concentrations of 1, 5, and 10 wt% and named optical biofilter_1%, optical biofilter_5%, and optical biofilter_10% (Note S3). All tests were conducted after water exchange that reached equilibrium in an air atmosphere of saturated salt water at ambient temperature. The cellulose film was fabricated by directly drying cellulose hydrogel at 97 °C using a rapid Köthen sheet former (RK-3A, FrankPTI, Vorchdorf, Austria). The generality of the biofilter preparation strategy was verified by using bamboo and hardwood (Notes S4 to S7).

### Characterizations

The surface and cross-profile morphology of samples were observed by scanning electron microscopy (SU8010, HITACHI Ltd., Tokyo, Japan) (5 kV) and confocal Raman spectroscopy (XploRA PLUS, Horiba Ltd., Japan). Molecular structures were determined by FTIR (Thermo Fisher Scientific Inc., USA) in the range of 650 to 4,000 cm^−1^ and 2-dimensional heteronuclear single quantum coherence (2D-HSQC) NMR (Bruker; 600 MHz). Fused lignin was hot-pressed for 1 h (180 °C, 5 MPa) for the 2D-HSQC NMR measurement. X-ray diffraction (Empyrean, PANalytical B.V., Almelo, Netherlands) with a Cu Kα monochromatic radiation source was used to characterize crystallization conditions with the 2*θ* angle from 5° to 40° and a scanning speed of 5°·min^−1^. Differential scanning calorimetry (DSC2500, TA Instruments, USA) was performed with a heating rate of 10 °C·min^−1^ in a nitrogen atmosphere; all samples were first heated at 105 °C for 10 min before testing from 30 to 200 °C. NIR diffuse reflectance spectra in the range of 4,000 to 10,000 cm^−1^ were obtained with an Fourier transform near-infrared analyzer (ANTARIS II, Thermo Fisher Scientific Inc., USA).

### Optical property testing

Transmittance and haze were measured by UV-Vis-NIR (UH-5700, HITACHI Ltd., Tokyo, Japan) with a wavelength range of 200 to 1,400 nm and a transmittance/haze tester (WGT-S, Shanghai Shenguang Industrial Co. Ltd., Shanghai, China) with a wavelength of 550 nm.

### Stability testing

Water vapor barrier properties were measured by the color change of silica gel in a sealed bottle (48 h) and calculated by the weight change of CaCl_2_ in a sealed bottle (120 h) in air atmosphere of saturated salt water (25 °C). The weight change of CaCl_2_ was used to calculate water vapor transmittance rate, equivalent to the ratio of the mass change of CaCl_2_ to the effective area for 1 d (in grams per square meter per day). Water absorption was measured by the weight before and after immersing cellulose film, lignin–cellulose film, and optical biofilter in deionized water for 30 min. Then, the shape stability was illustrated by recording the state of drying, wetting, and redrying. Water contact angle was measured using a contact angle measuring system (JC2000D1, Zhongchen Digital Technic Apparatus Co. Ltd., Shanghai, China) in 180 s at ambient temperature. Solvent stability was evaluated by immersing the optical biofilter for 21 d in deionized water, 90 vol% of aqueous acetic acid, dioxane, acetone, ethyl ether, *N*,*N*-dimethylformamide, ethanol, 1 wt% of aqueous NaOH, and *tert*-butanol. Thermal stability was measured by a thermogravimetric analyzer (SDT Q600, Thermal Advantage Inc., USA) from 100 to 700 °C with a heating rate of 20 °C·min^−1^ and a nitrogen atmosphere. Environmental stability of the optical biofilter with size of 20 mm × 20 mm was investigated in artificial soil environment and simulated environmental changes with deionized water and sun exposure for 150 d.

### Tensile strength

The tensile properties of cellulose film, lignin–cellulose film, and optical biofilter, 30 mm × 10 mm (length × width), were measured using an electromechanical universal testing machine [E43.104, MTS Systems (China) Co. Ltd.] equipped with a 250-N load cell and a constant speed of 5 mm·min^−1^.

## Data Availability

The data that support the findings of this study are available within this paper and/or included in the Supplementary Materials and from the corresponding author upon request. Source data are provided with this paper.

## Supplementary Materials

Supplementary 1 Figs. S1 to S35 Notes S1 to S7 Table S1 References [45–51]

## References

[1] Ghosh S, Cherumukkil S, Suresh CH, Ajayaghosh A. A supramolecular nanocomposite as a near-infrared-transmitting optical filter for security and forensic applications. Adv Mater. 2017;29(46):1703783. 10.1002/adma.201703783 29058799

[2] Muthu C, Pious JK, Seethal PS, Krishna N, Vijayakumar C. Formamidinium lead iodide perovskite nanocrystal/squaraine dye composite based visibly opaque and near-infrared transmitting material. Adv Opt Mater. 2020;8(24):2001130.

[3] Tian S, Tan J, Kang T, Cao C, Pan J, Xiao Y, Cui X, Li S, Lee CS. Harnessing polymer-matrix-mediated manipulation of intermolecular charge-transfer for near-infrared security applications. Adv Mater. 2022;34(38):2204749. 10.1002/adma.202204749 35862231

[4] Griebel JJ, Namnabat S, Kim ET, Himmelhuber R, Moronta DH, Chung WJ, Simmonds AG, Kim KJ, van der Laan J, Nguyen NA, et al. New infrared transmitting material via inverse vulcanization of elemental sulfur to prepare high refractive index polymers. Adv Mater. 2014;26(19):3014–3018. 24659231 10.1002/adma.201305607

[5] Anderson LE, Kleine TS, Zhang Y, Phan DD, Namnabat S, LaVilla EA, Konopka KM, Ruiz Diaz L, Manchester MS, Schwiegerling J, et al. Chalcogenide hybrid inorganic/organic polymers: Ultrahigh refractive index polymers for infrared imaging. ACS Macro Lett. 2017;6(5):500–504. 35610885 10.1021/acsmacrolett.7b00225

[6] Oliaei E, Olsen P, Lindstrom T, Berglund LA. Highly reinforced and degradable lignocellulose biocomposites by polymerization of new polyester oligomers. Nat Commun. 2022;13(1):5666. 36167843 10.1038/s41467-022-33283-z PMC9515094

[7] Xu T, Du H, Liu H, Liu W, Zhang X, Si C, Liu P, Zhang K. Advanced nanocellulose-based composites for flexible functional energy storage devices. Adv Mater. 2021;33(48):2101368. 10.1002/adma.202101368 PMC11468700 34561914

[8] Zhao X, Wang Y, Chen X, Yu X, Li W, Zhang S, Meng X, Zhao Z-M, Dong T, Anderson A, et al. Sustainable bioplastics derived from renewable natural resources for food packaging. Matter. 2023;6(1):97–127.

[9] Sun WB, Han ZM, Yue X, Zhang HY, Yang KP, Liu ZX, Li DH, Zhao YX, Ling ZC, Yang HB, et al. Nacre-inspired bacterial cellulose/mica nanopaper with excellent mechanical and electrical insulating properties by biosynthesis. Adv Mater. 2023;35(24):2300241. 10.1002/adma.202300241 36971025

[10] Jiang G, Wang G, Zhu Y, Cheng W, Cao K, Xu G, Zhao D, Yu H. A scalable bacterial cellulose ionogel for multisensory electronic skin. Research. 2022;2022:9814767. 35711672 10.34133/2022/9814767 PMC9188022

[11] Bai L, Li Q, Yang Y, Ling S, Yu H, Liu S, Li J, Chen W. Biopolymer nanofibers for nanogenerator development. Research. 2021;2021:1843061. 33709081 10.34133/2021/1843061 PMC7926511

[12] Zhu P, Yu Z, Sun H, Zheng D, Zheng Y, Qian Y, Wei Y, Lee J, Srebnik S, Chen W, et al. 3D printed cellulose nanofiber aerogel scaffold with hierarchical porous structures for fast solar-driven atmospheric water harvesting. Adv Mater. 2023;e2306653. 37696052 10.1002/adma.202306653

[13] Huang J, Yu L, Wang S, Qi L, Lu Z, Chen L, Xu D, Deng H, Chen C. An ultrathin nanocellulosic ion redistributor for long-life zinc anode. Innov Mater. 2023;1(2):100029.

[14] Wang J, Emmerich L, Wu J, Vana P, Zhang K. Hydroplastic polymers as eco-friendly hydrosetting plastics. Nat Sustain. 2021;4(10):877–883.

[15] Zhao D, Pang B, Zhu Y, Cheng W, Cao K, Ye D, Si C, Xu G, Chen C, Yu H. A stiffness-switchable, biomimetic smart material enabled by supramolecular reconfiguration. Adv Mater. 2022;34(10):2107857. 10.1002/adma.202107857 34964189

[16] Ye Y, Yu L, Lizundia E, Zhu Y, Chen C, Jiang F. Cellulose-based ionic conductor: An emerging material toward sustainable devices. Chem Rev. 2023;123(15):9204–9264. 37419504 10.1021/acs.chemrev.2c00618

[17] Zhou G, Zhang H, Su Z, Zhang X, Zhou H, Yu L, Chen C, Wang X. A biodegradable, waterproof, and thermally processable cellulosic bioplastic enabled by dynamic covalent modification. Adv Mater. 2023;35(25):2301398. 10.1002/adma.202301398 37127887

[18] Liu C, Luan P, Li Q, Cheng Z, Sun X, Cao D, Zhu H. Biodegradable, hygienic, and compostable tableware from hybrid sugarcane and bamboo fibers as plastic alternative. Matter. 2020;3(6):2066–2079.

[19] Ou J, Hu S, Yao L, Chen Y, Qi H, Yue F. Simultaneous strengthening and toughening lignin/cellulose nanofibril composite films: Effects from flexible hydrogen bonds. Chem Eng J. 2023;453: Article 139770.

[20] Jia D, Xie J, Dirican M, Fang D, Yan C, Liu Y, Li C, Cui M, Liu H, Chen G, et al. Highly smooth, robust, degradable and cost-effective modified lignin-nanocellulose green composite substrates for flexible and green electronics. Compos Part B Eng. 2022;236: Article 109803.

[21] Sadeghifar H, Venditti R, Jur J, Gorga RE, Pawlak JJ. Cellulose-lignin biodegradable and flexible UV protection film. ACS Sustain Chem Eng. 2016;5(1):625–631.

[22] Wang S, Li L, Zha L, Koskela S, Berglund LA, Zhou Q. Wood xerogel for fabrication of high-performance transparent wood. Nat Commun. 2023;14(1):2827. 37198187 10.1038/s41467-023-38481-x PMC10192348

[23] Guan Q, Yang K, Han Z, Yang H, Ling Z, Yin C, Yu S. Sustainable multiscale high-haze transparent cellulose fiber film via a biomimetic approach. ACS Mater Lett. 2021;4:87–92.

[24] Li K, Wang S, Chen H, Yang X, Berglund LA, Zhou Q. Self-densification of highly mesoporous wood structure into a strong and transparent film. Adv Mater. 2020;32(42):2003653. 10.1002/adma.202003653 32881202

[25] Kaschuk JJ, Al Haj Y, Rojas OJ, Miettunen K, Abitbol T, Vapaavuori J. Plant-based structures as an opportunity to engineer optical functions in next-generation light management. Adv Mater. 2022;34(6):2104473. 10.1002/adma.202104473 34699648

[26] Zhang Y, Naebe M. Lignin: A review on structure, properties, and applications as a light-colored UV absorber. ACS Sustain Chem Eng. 2021;9(4):1427–1442.

[27] Zhang Y, Wei Y, Qian Y, Zhang M, Zhu P, Chen G. Lignocellulose enabled highly transparent nanopaper with tunable ultraviolet-blocking performance and superior durability. ACS Sustain Chem Eng. 2020;8(46):17033–17041.

[28] Yang W, Jiao L, Liu W, Deng Y, Dai H. Morphology control for tunable optical properties of cellulose nanofibrils films. Cellulose. 2018;25(10):5909–5918.

[29] Moreno A, Delgado-Lijarcio J, Ronda JC, Cadiz V, Galia M, Sipponen MH, Lligadas G. Breathable lignin nanoparticles as reversible gas swellable nanoreactors. Small. 2023;19(7):2205672. 10.1002/smll.202205672 36478382

[30] Schneider WDH, Dillon AJP, Camassola M. Lignin nanoparticles enter the scene: A promising versatile green tool for multiple applications. Biotechnol Adv. 2021;47: Article 107685. 33383155 10.1016/j.biotechadv.2020.107685

[31] Kubo S, Uraki Y, Sano Y. Preparation of carbon fibers from softwood lignin by atmospheric acetic acid pulping. Carbon. 1998;36:1119–1124.

[32] Buono P, Duval A, Verge P, Averous L, Habibi Y. New insights on the chemical modification of lignin: Acetylation versus silylation. ACS Sustain Chem Eng. 2016;4(10):5212–5222.

[33] Guterman R, Molinari V, Josef E. Ionic liquid lignosulfonate: Dispersant and binder for preparation of biocomposite materials. Angew Chem Int Ed Engl. 2019;58(37):13044–13050. 31268204 10.1002/anie.201907385 PMC7687102

[34] Xia Q, Chen C, Yao Y, Li J, He S, Zhou Y, Li T, Pan X, Yao Y, Hu L. A strong, biodegradable and recyclable lignocellulosic bioplastic. Nat Sustain. 2021;4(7):627–635.

[35] Fang S, Lyu X, Tong T, Lim AI, Li T, Bao J, Hu YH. Turning dead leaves into an active multifunctional material as evaporator, photocatalyst, and bioplastic. Nat Commun. 2023;14(1):1203. 36864061 10.1038/s41467-023-36783-8 PMC9981597

[36] De Santi A, Monti S, Barcaro G, Zhang Z, Barta K, Deuss PJ. New mechanistic insights into the lignin β-O-4 linkage acidolysis with ethylene glycol stabilization aided by multilevel computational chemistry. ACS Sustain Chem Eng. 2021;9(5):2388–2399. 33585085 10.1021/acssuschemeng.0c08901 PMC7874265

[37] Qu X, Zhao Y, Chen Z, Wang S, Ren Y, Wang Q, Shao J, Wang W, Dong X. Thermoresponsive lignin-reinforced poly(ionic liquid) hydrogel wireless strain sensor. Research. 2021;2021:9845482. 34957404 10.34133/2021/9845482 PMC8674648

[38] Li Y, Zhao S, Hu D, Ragauskas AJ, Cao D, Liu W, Si C, Xu T, Zhao P, Song X, et al. Role evaluation of active groups in lignin on UV-shielding performance. ACS Sustain Chem Eng. 2022;10(36):11856–11866.

[39] Xia Q, Chen C, Li T, He S, Gao J, Wang X, Hu L. Solar-assisted fabrication of large-scale, patternable transparent wood. Sci Adv. 2021;7:eabd7342. 33571122 10.1126/sciadv.abd7342 PMC7840122

[40] Zhu M, Li T, Davis CS, Yao Y, Dai J, Wang Y, AlQatari F, Gilman JW, Hu L. Transparent and haze wood composites for highly efficient broadband light management in solar cells. Nano Energy. 2016;26:332–339.

[41] Jiang Y, Wang Z, Liu X, Yang Q, Huang Q, Wang L, Dai Y, Qin C, Wang S. Highly transparent, UV-shielding, and water-resistant lignocellulose nanopaper from agro-industrial waste for green optoelectronics. ACS Sustain Chem Eng. 2020;8(47):17508–17519.

[42] Jiang B, Chen C, Liang Z, He S, Kuang Y, Song J, Mi R, Chen G, Jiao M, Hu L. Lignin as a wood-inspired binder enabled strong, water stable, and biodegradable paper for plastic replacement. Adv Funct Mater. 2019;30(4):1906307.

[43] Oliaei E, Berthold F, Berglund LA, Lindström T. Eco-friendly high-strength composites based on hot-pressed lignocellulose microfibrils or fibers. ACS Sustain Chem Eng. 2021;9(4):1899–1910.

[44] Chen X, Huang Y, Zhang L, Liu J, Wang C, Wu M. Cellulose nanofiber assisted dispersion of hydrophobic SiO_2_ nanoparticles in water and its superhydrophobic coating. Carbohydr Polym. 2022;290: Article 119504. 35550757 10.1016/j.carbpol.2022.119504

[45] Yu S, Gan M, Chen Y, Hu Z, Xie Y, Feng Q. Fabrication of lignin-containing cellulose bio-composite based on unbleached corncob and wheat straw pulp. Int J Biol Macromol. 2022;208:741–747. 35367472 10.1016/j.ijbiomac.2022.03.192

[46] Li J, Zhang X, Zhang J, Mi Q, Jia F, Wu J, Yu J, Zhang J. Direct and complete utilization of agricultural straw to fabricate all-biomass films with high-strength, high-haze and UV-shielding properties. Carbohydr Polym. 2019;223: Article 115057. 31427002 10.1016/j.carbpol.2019.115057

[47] Shu L, Zhang XF, Wang Z, Yao J. Structure reorganization of cellulose hydrogel by green solvent exchange for potential plastic replacement. Carbohydr Polym. 2022;275: Article 118695. 34742422 10.1016/j.carbpol.2021.118695

[48] Oliaei E, Lindén PA, Wu Q, Berthold F, Berglund L, Lindström T. Microfibrillated lignocellulose (MFLC) and nanopaper films from unbleached Kraft softwood pulp. Cellulose. 2019;27(4):2325–2341.

[49] Jiang Y, Wang Z, Zhou L, Jiang S, Liu X, Zhao H, Huang Q, Wang L, Chen G, Wang S. Highly efficient and selective modification of lignin towards optically designable and multifunctional lignocellulose nanopaper for green light-management applications. Int J Biol Macromol. 2022;206:264–276. 35240206 10.1016/j.ijbiomac.2022.02.147

[50] Fang Z, Zhu H, Bao W, Preston C, Liu Z, Dai J, Li Y, Hu L. Highly transparent paper with tunable haze for green electronics. Energy Environ Sci. 2014;7(10):3313–3319.

[51] Li Y, Fu Q, Rojas R, Yan M, Lawoko M, Berglund L. Lignin-retaining transparent wood. ChemSusChem. 2017;10(17):3445–3451. 28719095 10.1002/cssc.201701089 PMC5601211

