# Supplementary material for: Lignocellulose-Based Optical Biofilter with High Near-Infrared Transmittance via Lignin Capturing–Fusing Approach
Source: Research (Wash D C). 2023 Oct 16;6:0250. doi: 10.34133/research.0250 (PMC10585486; doi:10.34133/research.0250)
Supplement: Supplementary 1 — Figs. S1 to S35 Notes S1 to S7 Table S1 References [45–51] [file research.0250.f1.docx]

# **Supplementary Figures**


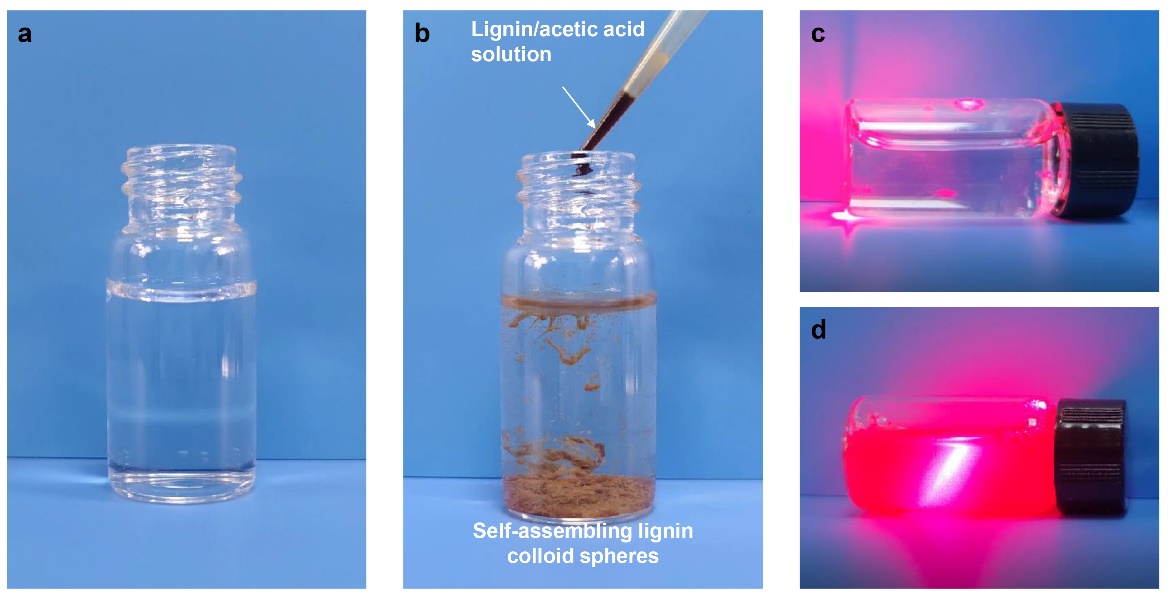


**Fig. S1.** Tyndall effect caused by lignin colloidal spheres. (a) Deionized water. (b) Lignin solvent/antisolvent assembly in deionized water. (c-d) Comparation of Tyndall effect in deionized water (c) and lignin colloid spheres suspension (d).

Because a clear light path was produced in the lignin colloidal sphere suspension, we speculated that self-assembly of lignin occurred during transfer from acetic acid to deionized water.


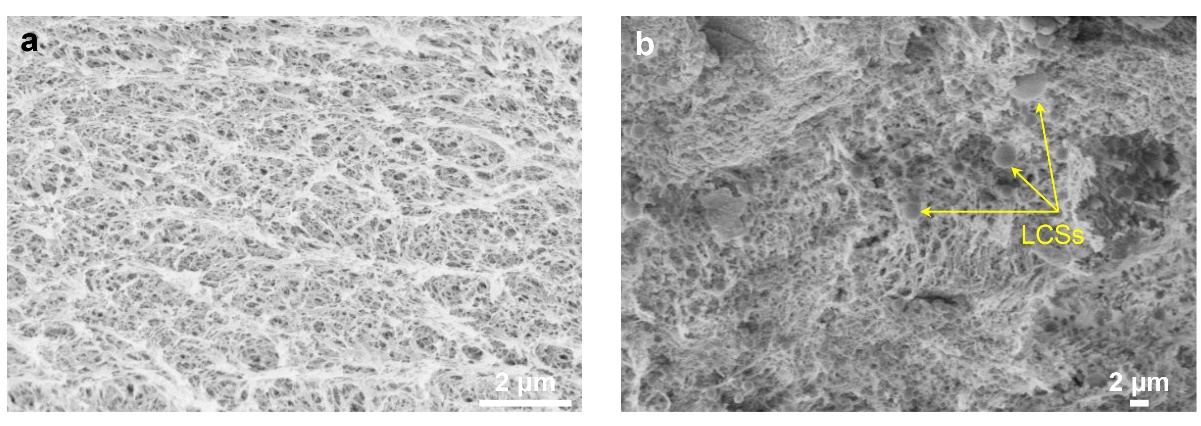


**Fig. S2.** SEM images of cellulose hydrogel cross-section (a) and lignin-cellulose hydrogel (b) treated with tertiary butanol. LCSs: lignin colloid spheres.


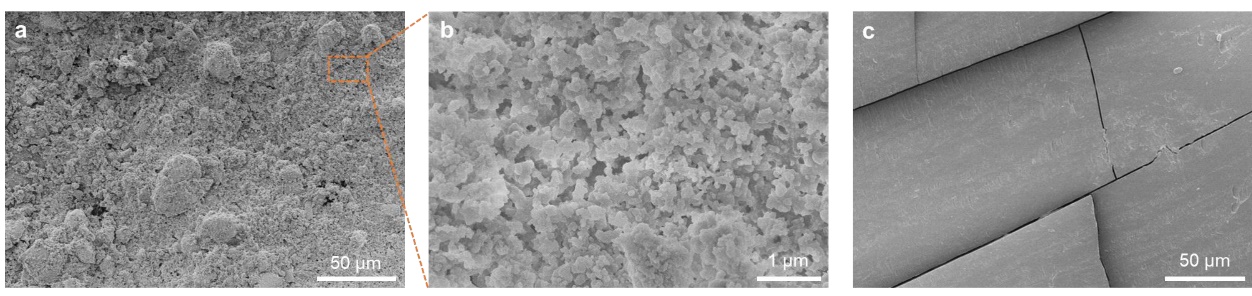


**Fig. S3.** SEM images of lignin (a-b) and fused lignin (c).


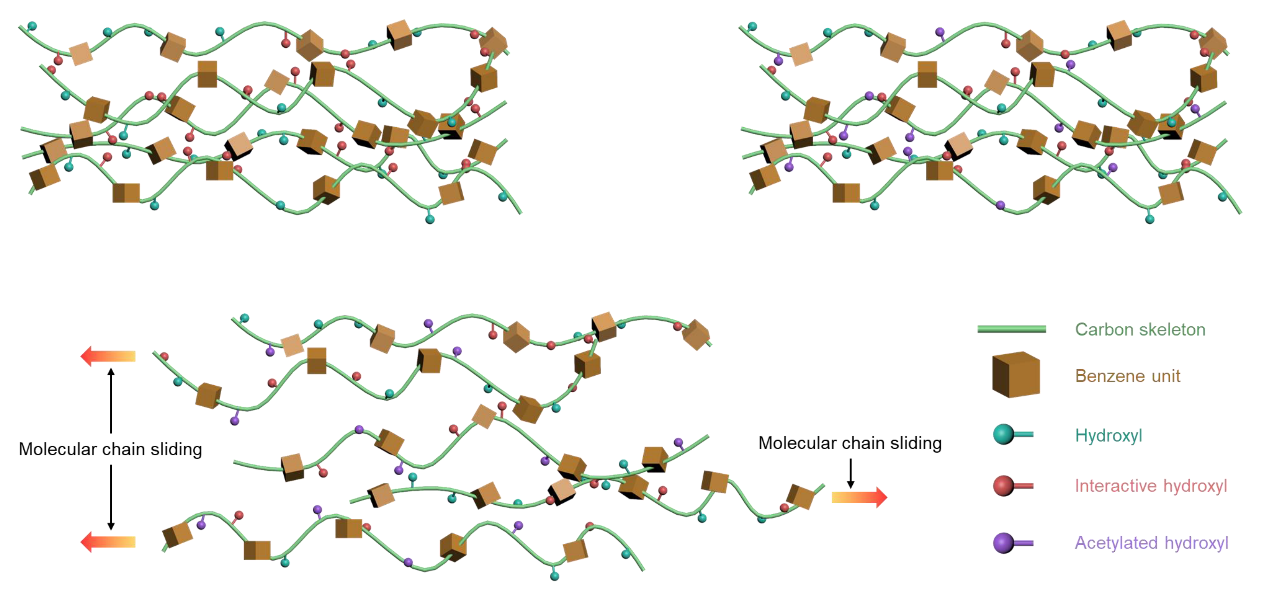


**Fig. S4.** Partial acetylation of hydroxyl groups on the lignin chains prevents hydroxyl groups from forming hydrogen bonds, massively disrupting the forces between lignin chains and increasing mobility at high temperature.


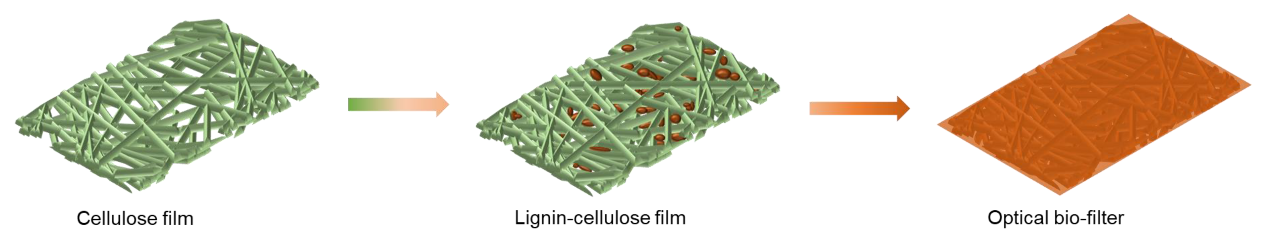


**Fig. S5.** Schematic of internal structural changes.

According to the properties of lignin self-assembly and fusion, we developed this lignin capturing-fusing approach. A cellulose network without lignin exposes many pores. Lignin self-assembling colloidal spheres initially fill these pores after drying, but abundant gaps remain. After hot pressing, the fused lignin fills the gaps and holds the cellulose fibers tightly, creating a homogenous dense structure.


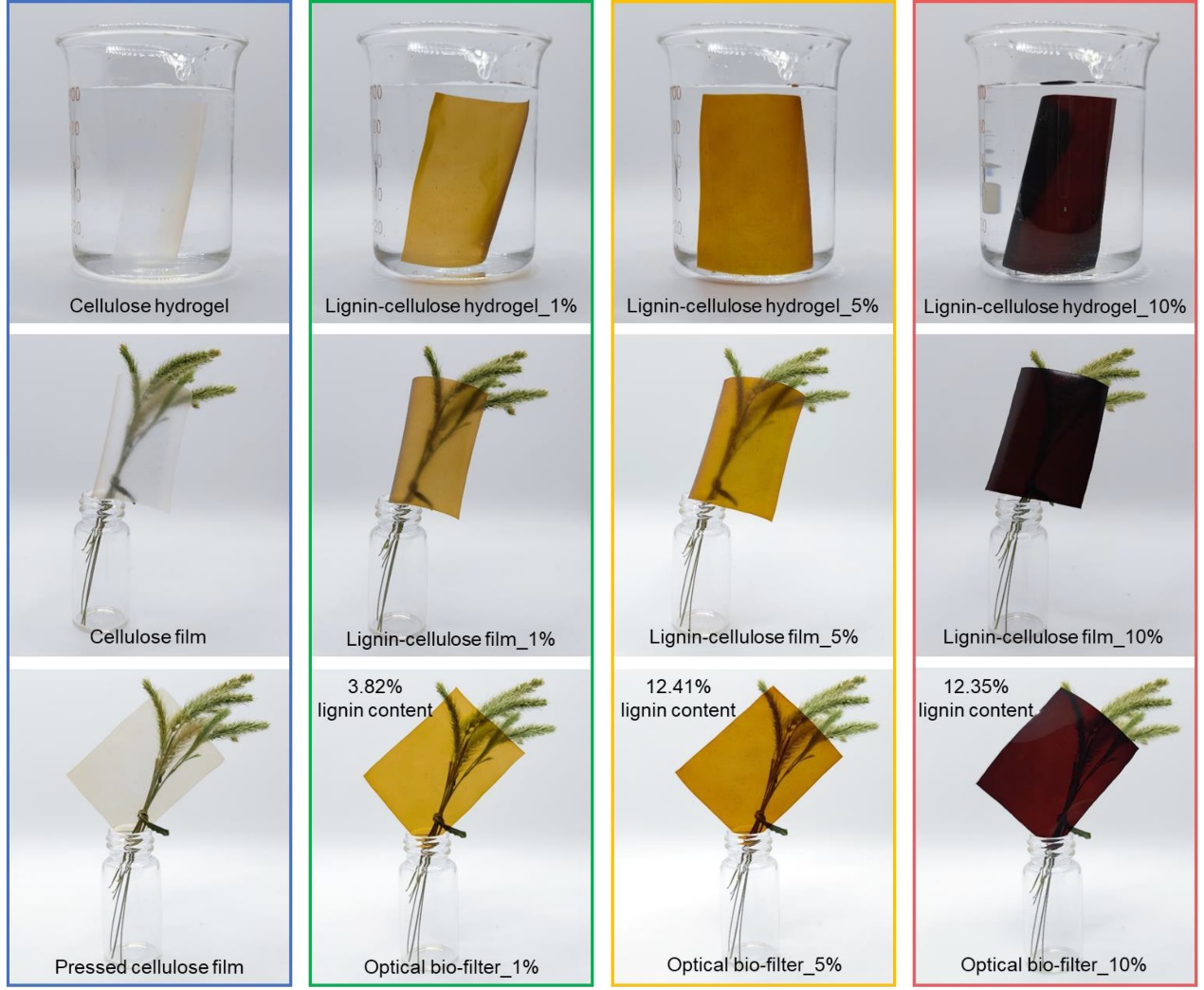


**Fig. S6.** Digital images of cellulose hydrogel, lignin-cellulose hydrogels with different concentrations of lignin, cellulose film, lignin-cellulose films with different concentrations of lignin, pressed cellulose film and optical bio-filters with different concentrations of lignin.


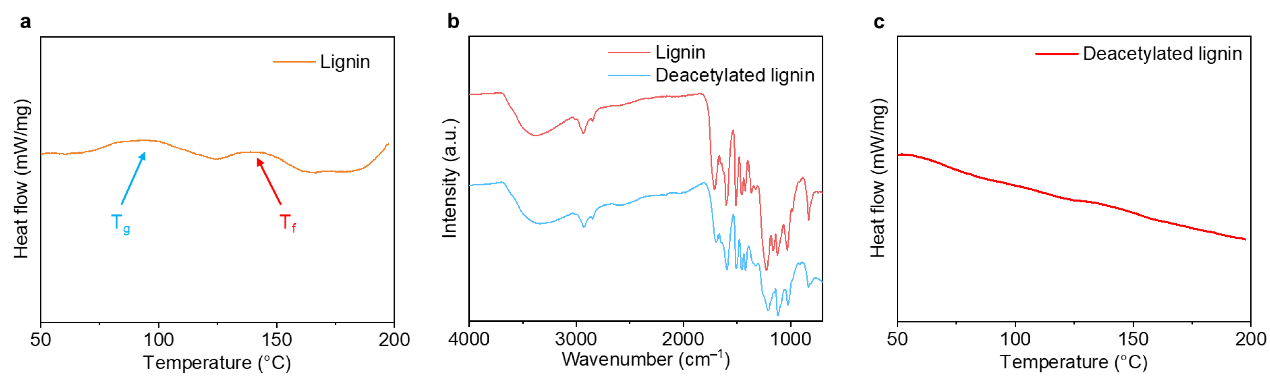


**Fig. S7.** (a) Differential scanning calorimetry (DSC) of acetic acid lignin from corncob. (b) Fourier transform infrared (FTIR) spectroscopy of acetic acid lignin from corncob and deacetylated lignin. (c) DSC curve of deacetylated lignin.

After comparing the DSC curve of acetic acid lignin with the curve for deacetylated lignin, we found that the peaks of the deacetylated lignin became almost absent, which indicated the unique thermal properties of acetic acid lignin and the importance of acetylation.

**Deacetylation of corncob acetic acid lignin with aqueous NaOH：**

Ten grams corncob acetic acid lignin powder was dispersed in 100 mL aqueous NaOH (1 mol/L); the mixed lignin/NaOH solution was stirred for 24 hours at ambient conditions for complete deacetylation followed by neutralization with HCl. Acetic acid was then added to assist the deposition and isolation of lignin according to Note S1.


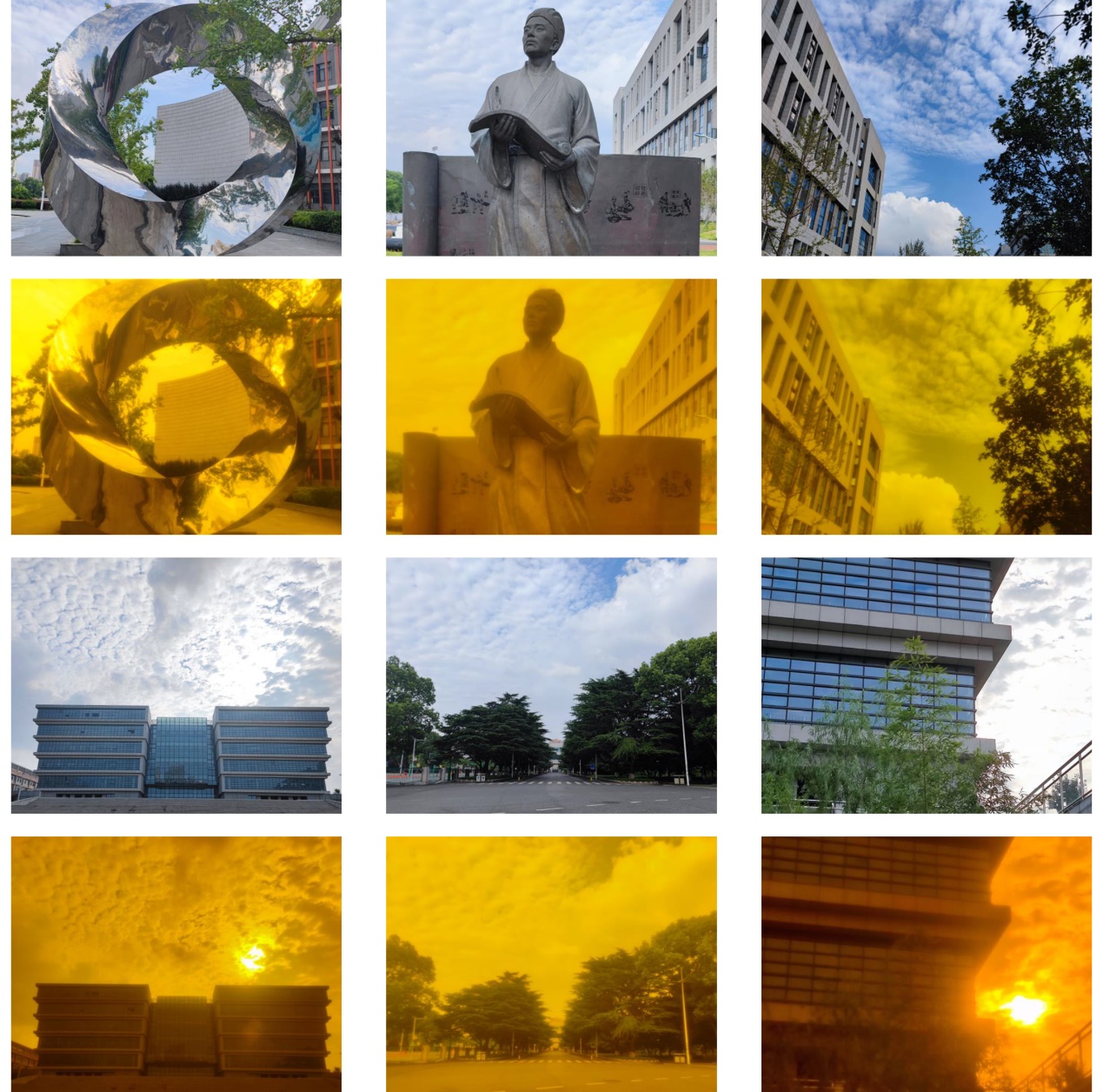


**Fig. S8.** Digital images with and without the optical bio-filter on the camera lens.


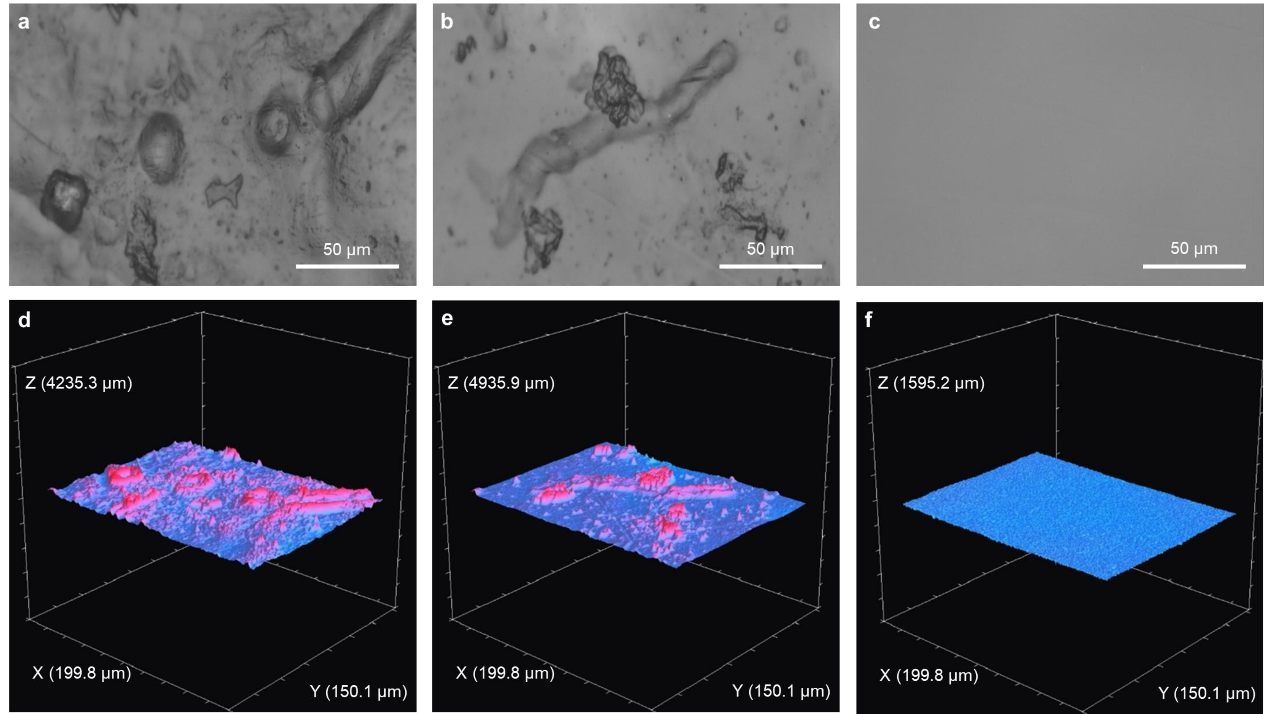


**Fig. S9.** 2D and 3D Raman surface imaging of cellulose film (a, d), lignin-cellulose film (b, e), and optical bio-filter (c, f).


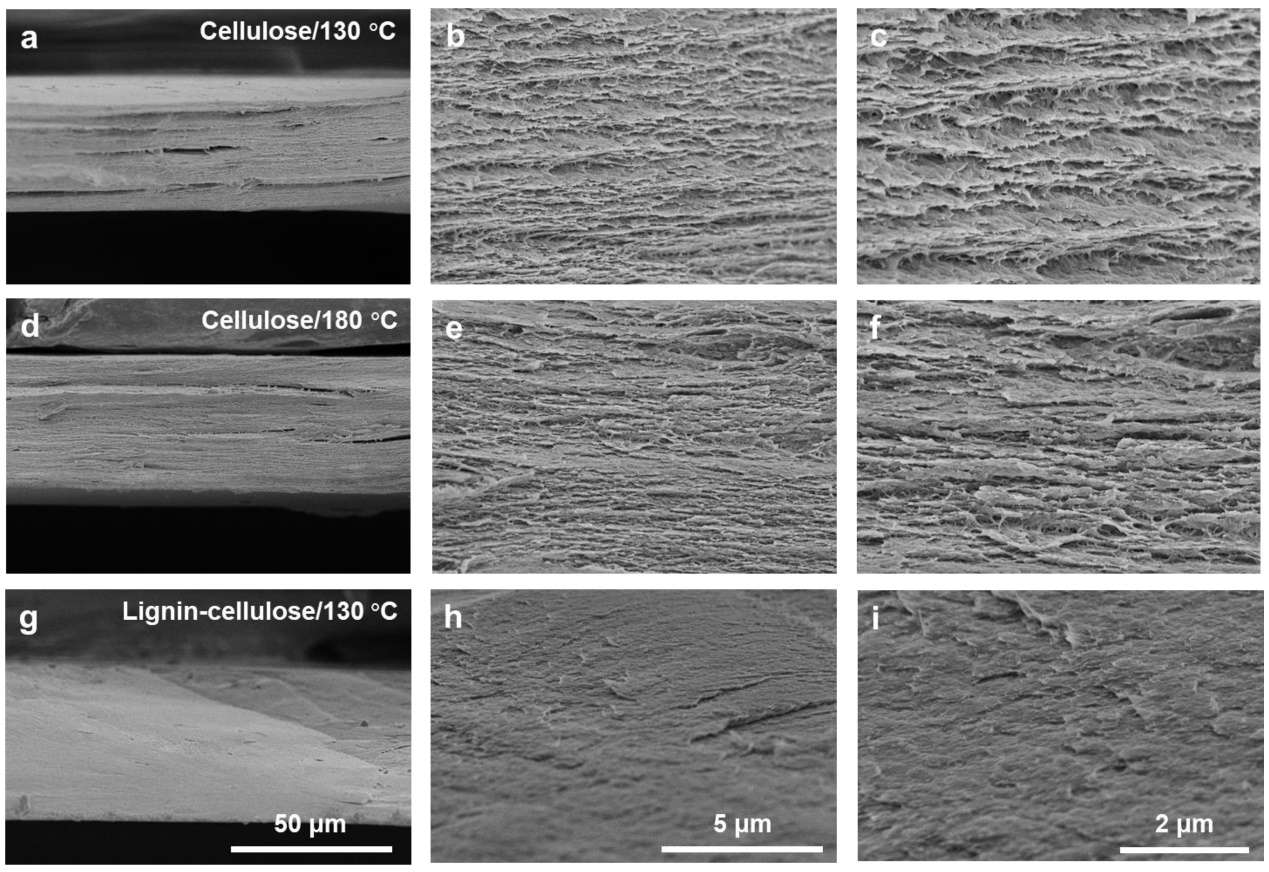


**Fig. S10.** (a-f) SEM images of cellulose film hot-pressed at 130 °C (a-c) and 180 °C (d-f). (g-i) SEM images of lignin-cellulose film hot-pressed at 130 °C.


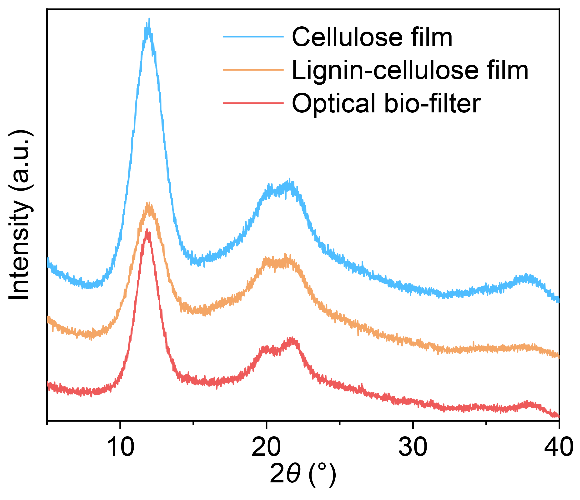


**Fig. S11.** X-ray diffraction patterns of cellulose film, lignin-cellulose film, and optical bio-filter.


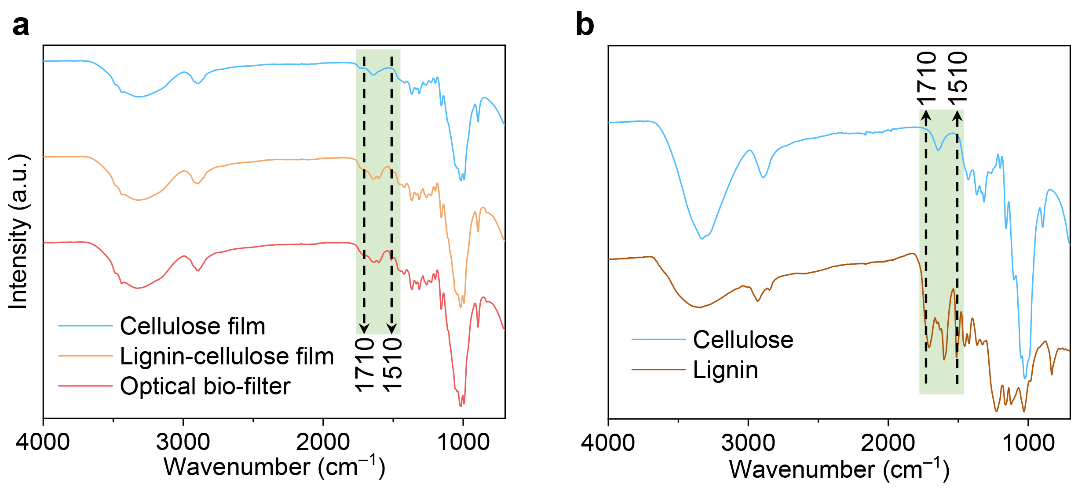


**Fig. S12.** (a) FTIR spectra of cellulose film, lignin-cellulose film, and optical bio-filter. (b) FTIR spectra of cellulose powder and lignin powder.


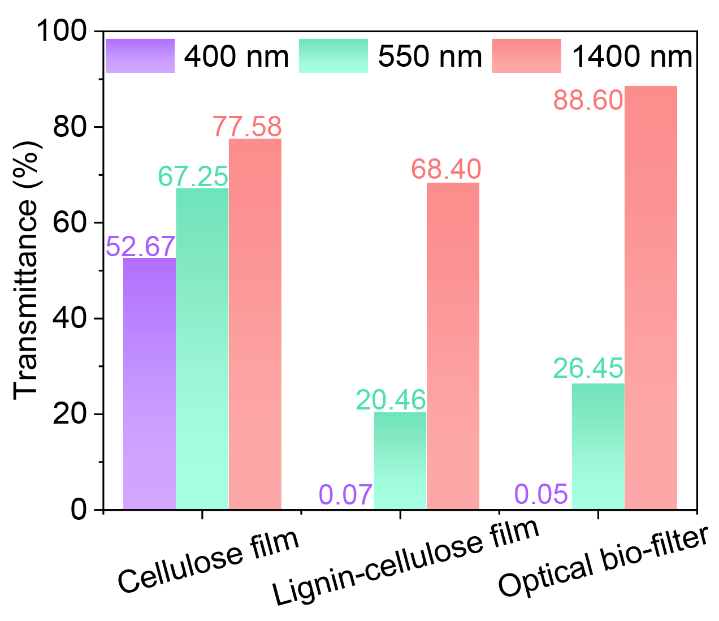


**Fig. S13.** Transmittance of cellulose film, lignin-cellulose film, and optical bio-filters at 400 nm, 550 nm, and 1400 nm.


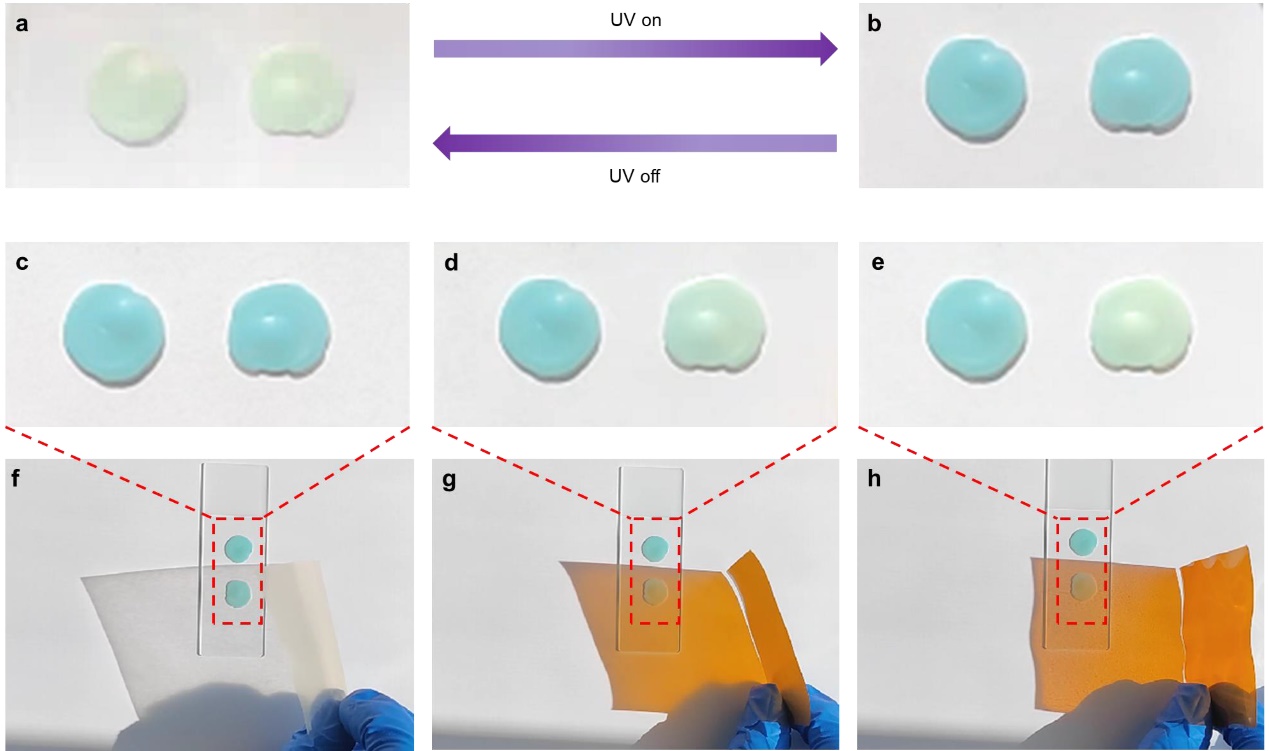


**Fig. S14.** (a-b) Images of UV-responsive paint in normal state (a) and color-changed state (b). (c-h) UV-shielding properties of cellulose film (c, f), lignin-cellulose film (d, g), and optical bio-filter (e, h).

UV-responsive paint changes color upon absorption of UV light. When UV light from the sun was blocked by a lignin-cellulose film or optical bio-filter, a color change did not occur, which indicated effective UV-blocking by the films.


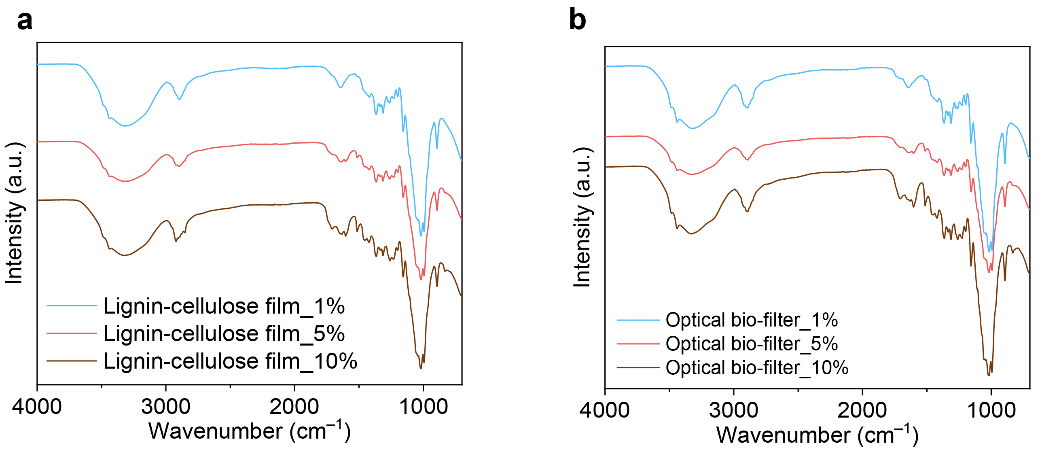


**Fig. S15.** FTIR spectra of lignin-cellulose films with different concentrations of lignin (a) and optical bio-filters with different concentrations of lignin (b).


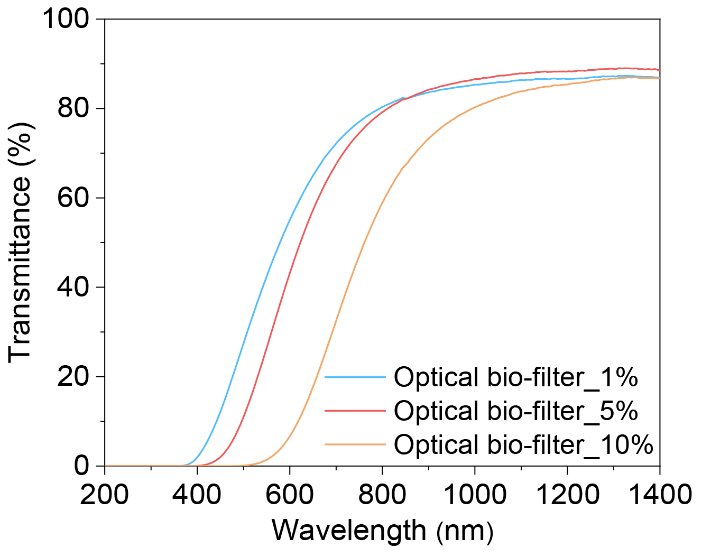


**Fig. S16.** Transmittance from 200 nm to 1400 nm of optical bio-filters with different concentrations of lignin.

The transmittance of optical bio-filters with different lignin content showed that a trace amount of lignin was enough to filter out almost all UV-light. More importantly, by varying the lignin content, we achieved a selective transmittance in the visible region and maintained high NIR transmittance at the same time, which enabled us to design different optical bio-filters for different applications.


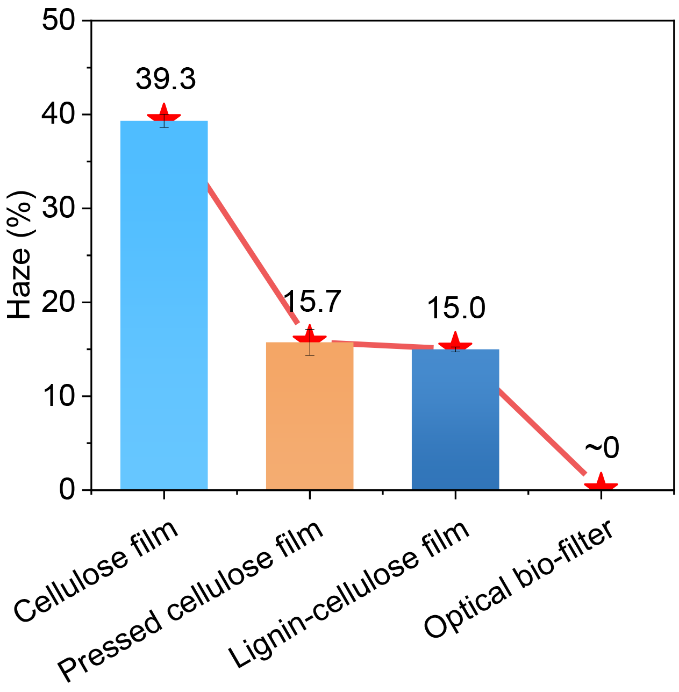


**Fig. S17.** Haze of cellulose film, pressed cellulose film, lignin-cellulose film, and optical bio-filter at 550 nm.

The four groups show that hot-pressing reduced haze attributed to smoothed surface and dense structure, but many defective structures remained that needed elimination by fused lignin.


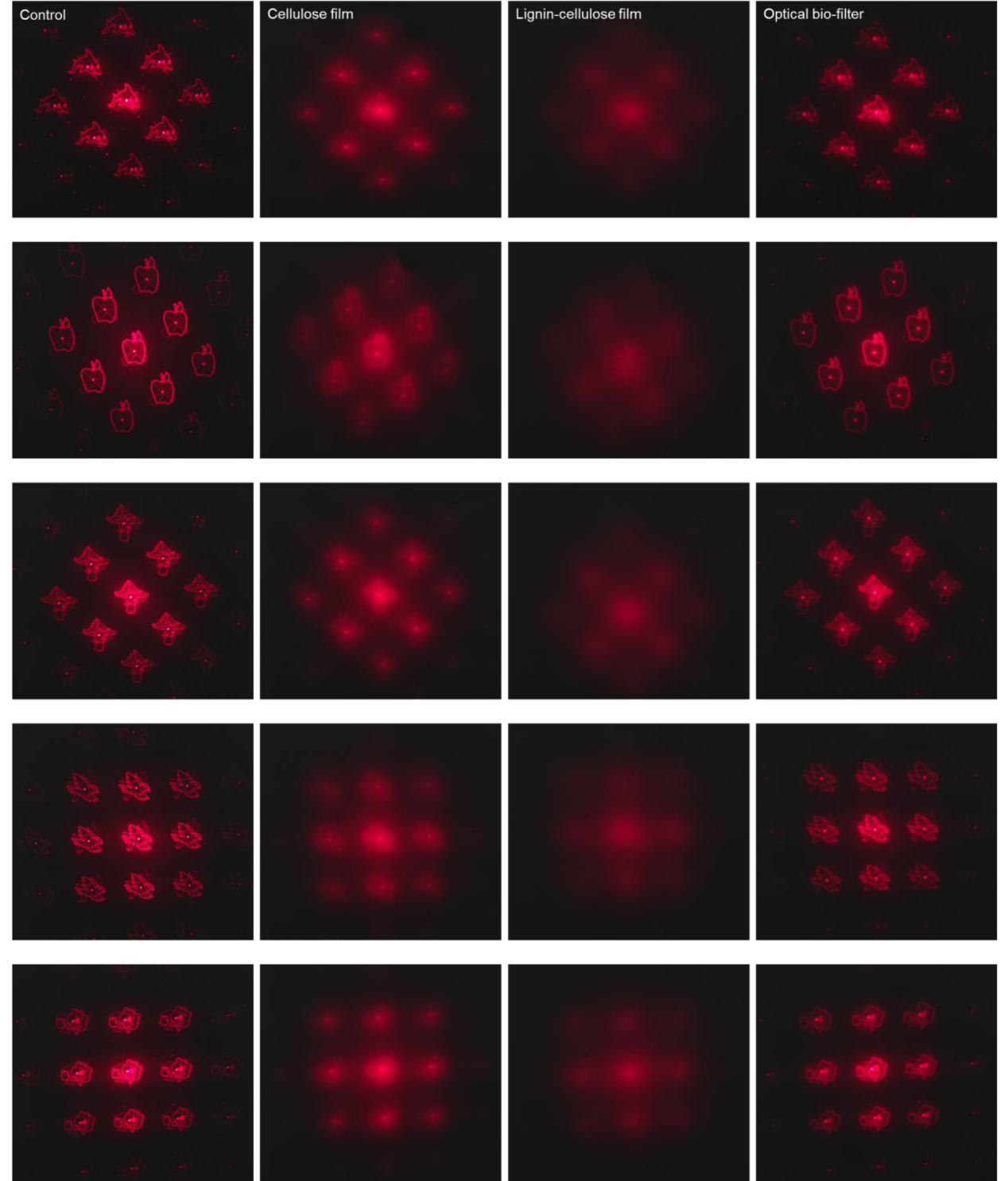


**Fig. S18.** Pictures of red laser pointer passing through cellulose film, lignin-cellulose film, and optical bio-filter.


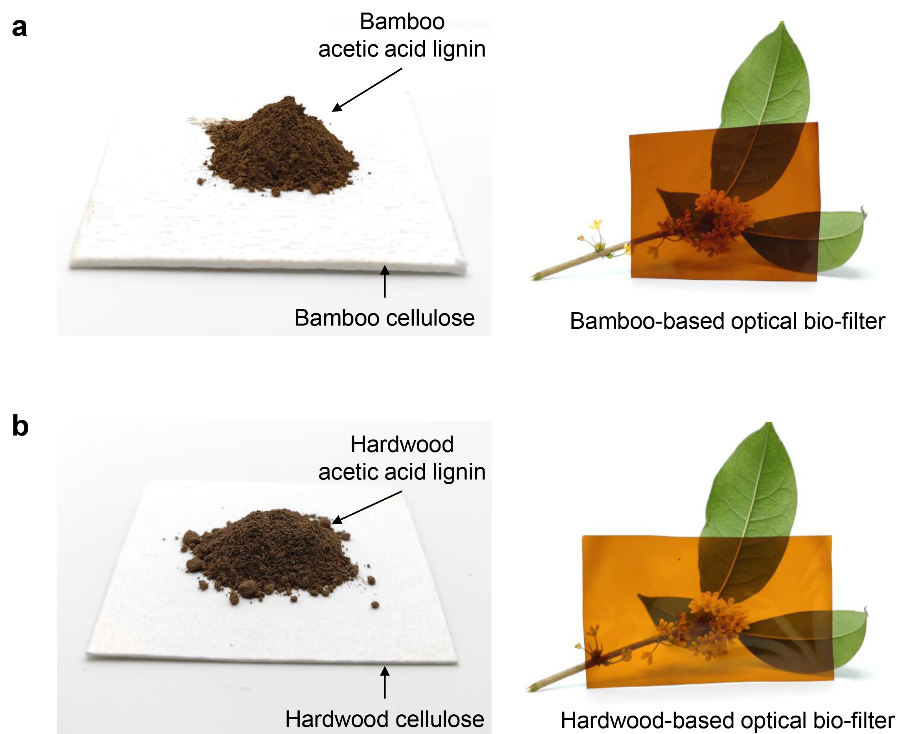


**Fig. S19.** Generality of optical bio-filters process using bamboo (a) and hardwood (b).

To assess the generality of our approach, we conducted the same experiments using bamboo and wood. Optical bio-filters prepared from those materials had the same transparency as filters prepared from corncob.


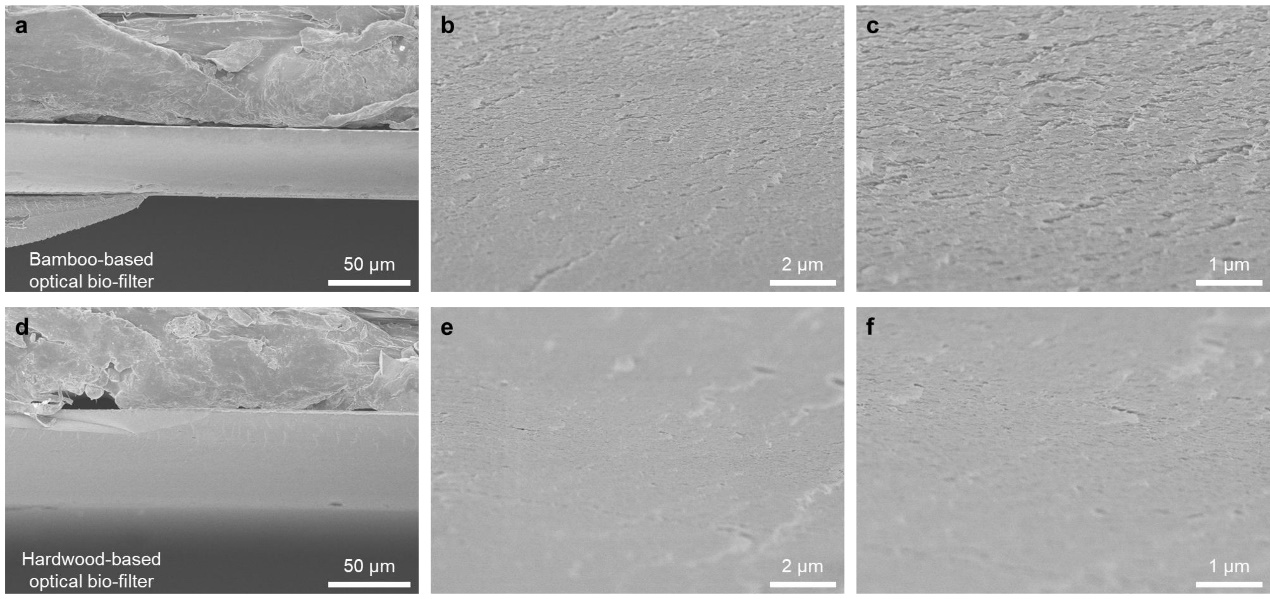


**Fig. S20.** SEM images of optical bio-filters fabricated with bamboo (a-c) and hardwood (d-f).


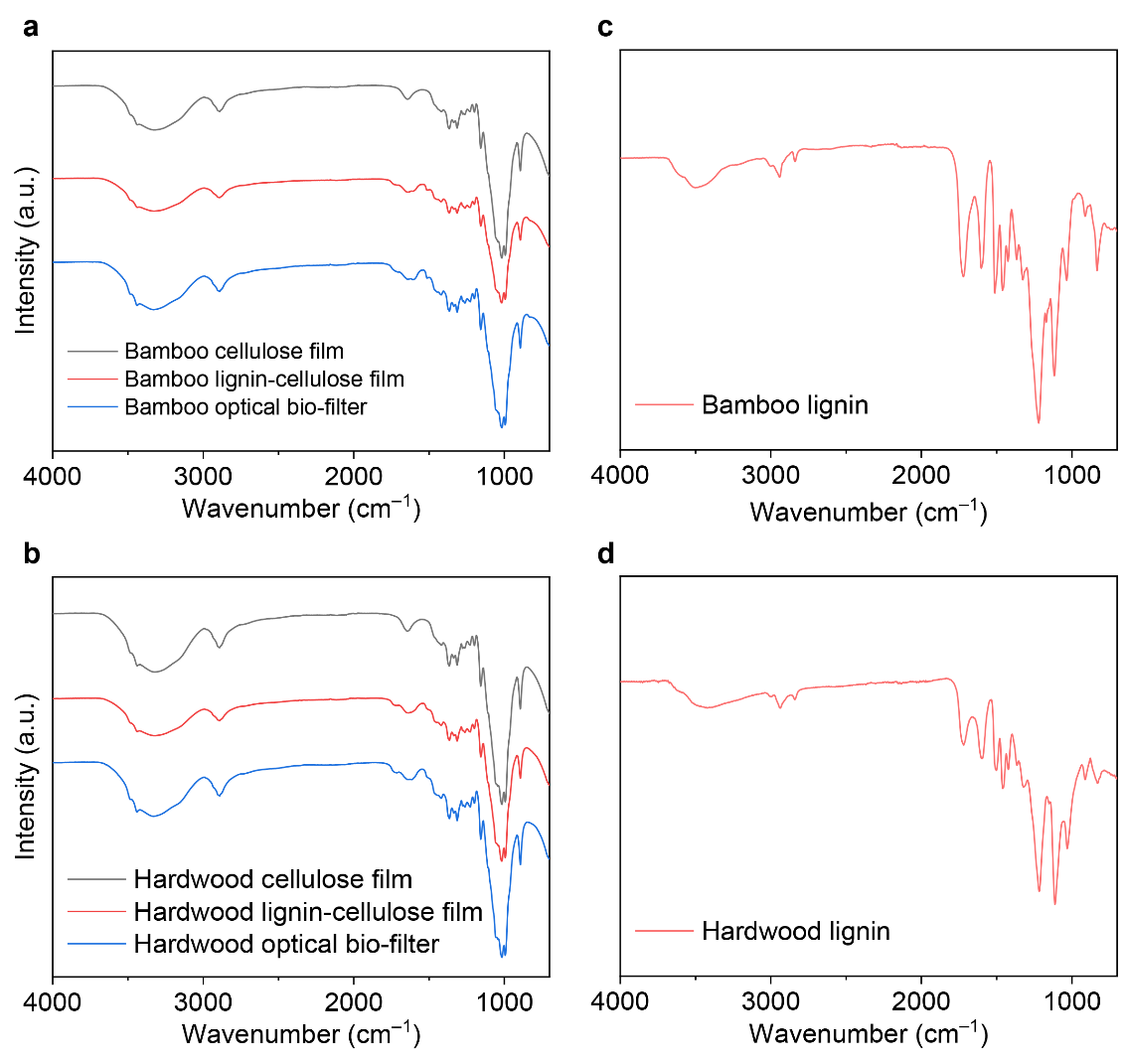


**Fig. S21.** (a-b) FTIR spectra of cellulose films, lignin-cellulose films, and optical bio-filters fabricated using bamboo (a) and hardwood (b). (c-d) FTIR spectra of acetic acid lignin from bamboo (c) and hardwood (d).


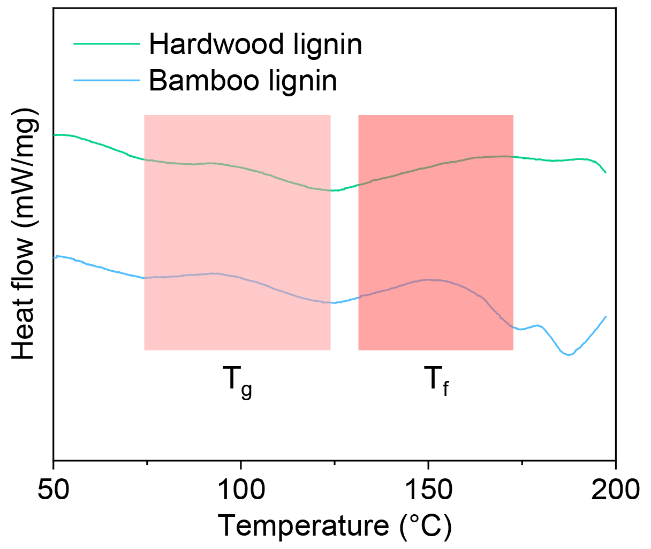


**Fig. S22.** DSC curves of hardwood acetic acid lignin and bamboo acetic acid lignin.


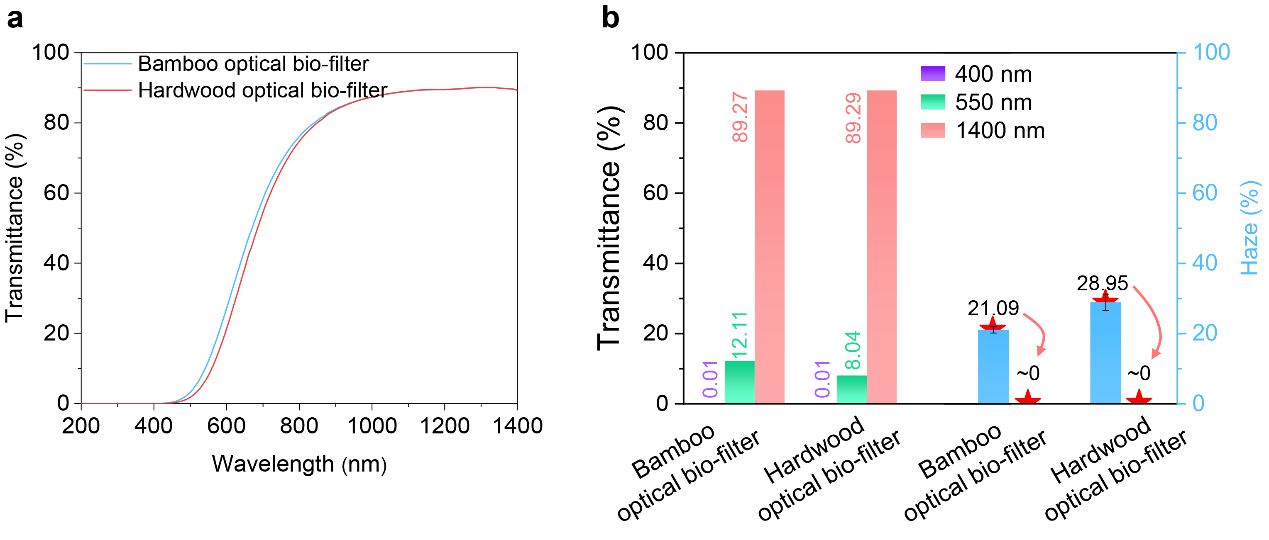


**Fig. S23.** (a) Transmittance from 200 nm to 1400 nm of optical bio-filters fabricated with bamboo and hardwood. (b) Transmittance at 400 nm, 550 nm, and 1400 nm and haze at 550 nm of optical bio-filters fabricated with bamboo and hardwood.


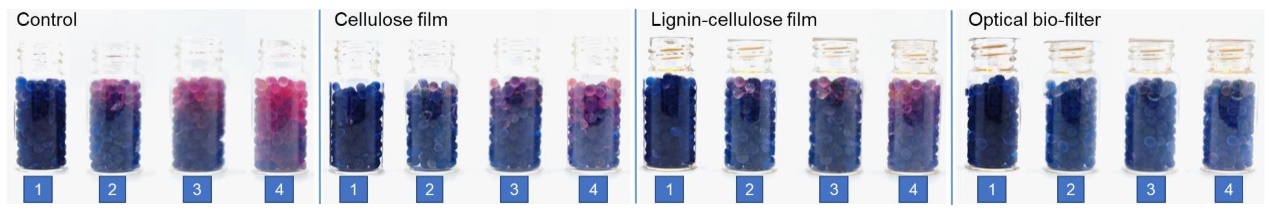


**Fig. S24.** Moisture barrier properties of cellulose film, lignin-cellulose film, and optical bio-filter using silica gel in sealed bottles. 1, 0h; 2, 12h; 3, 24 h; 4, 48 h.


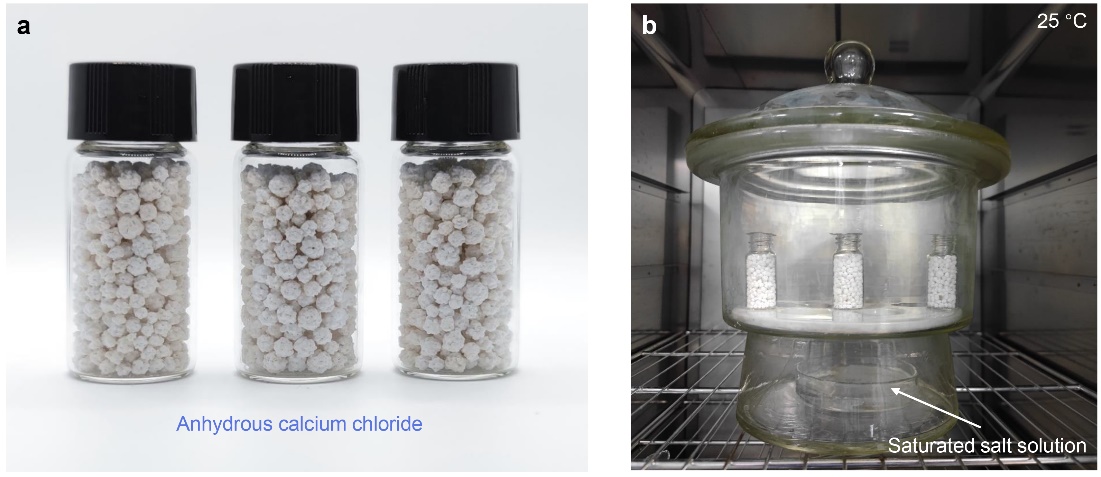


**Fig. S25.** Measurement of water vapor transmission rate using CaCl_2_ in air atmosphere of saturated salt water.


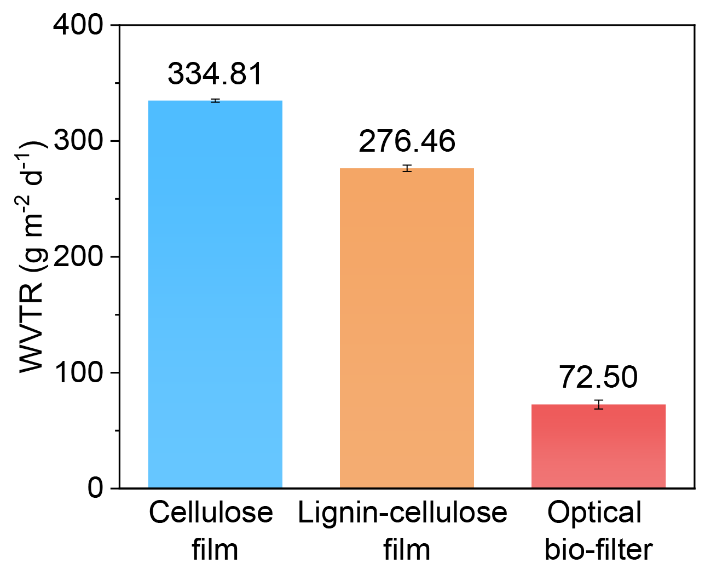


**Fig. S26.** Water vapor transmission rate of cellulose film, lignin-cellulose film, and optical bio-filter.


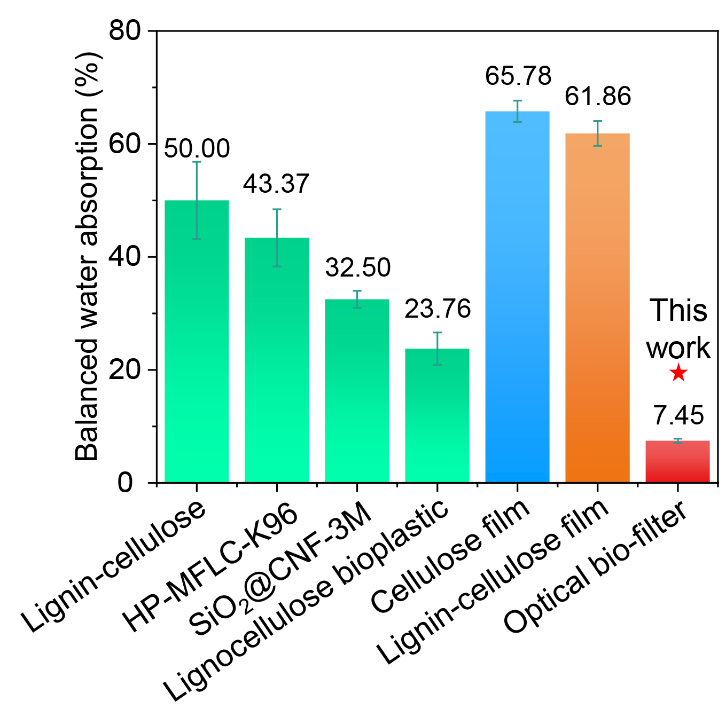


**Fig. S27.** Balanced water absorption of optical bio-filter compared with other lignocellulose-based materials.


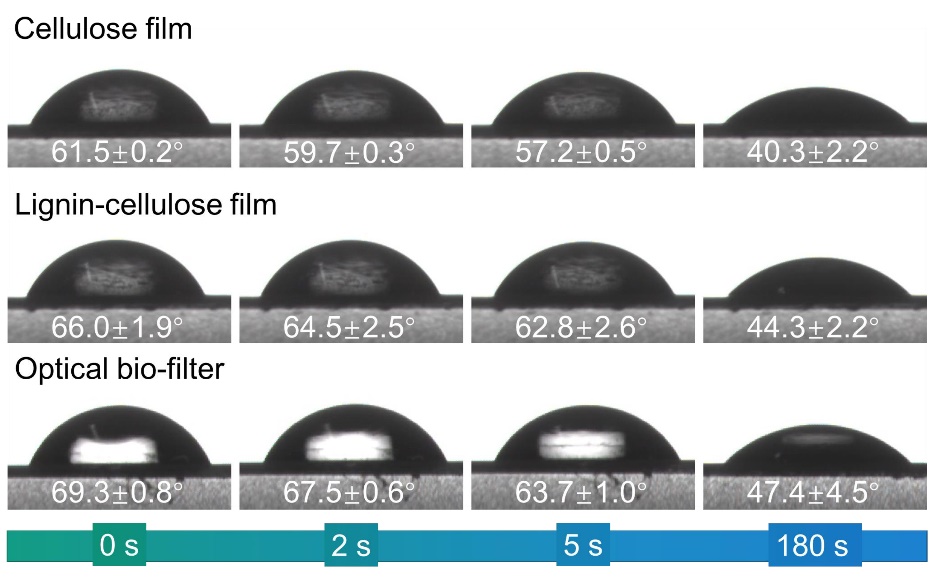


**Fig. S28.** Water contact angle of cellulose film, lignin-cellulose film, and optical bio-filter.


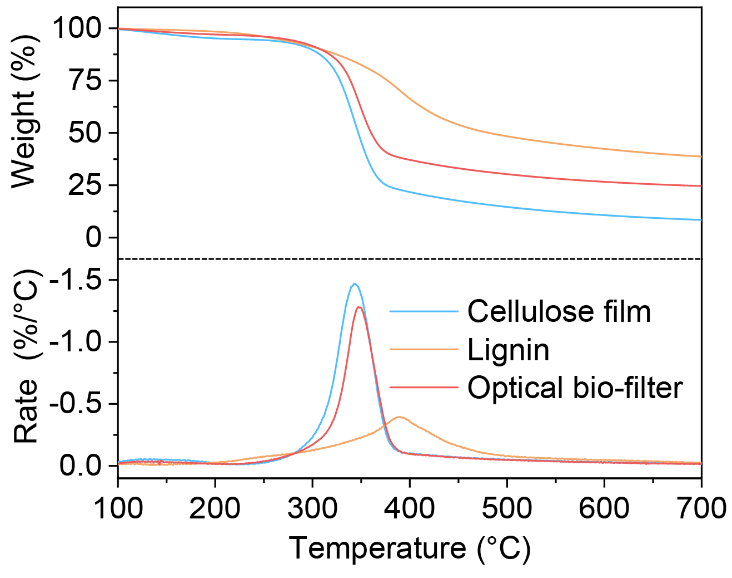


**Fig. S29.** Thermogravimetry (TG) and derivative thermogravimetry (DTG) curves of cellulose film, acetic acid lignin from corncob, and optical bio-filter.


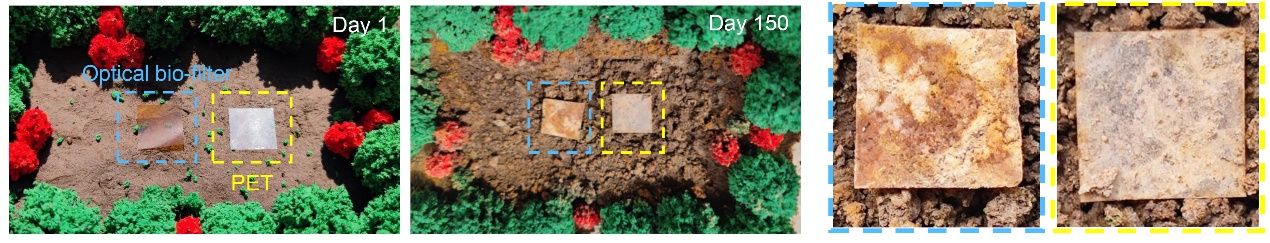


**Fig. S30.** Environmental stability of optical bio-filter compared to PET plastic for 150 days.


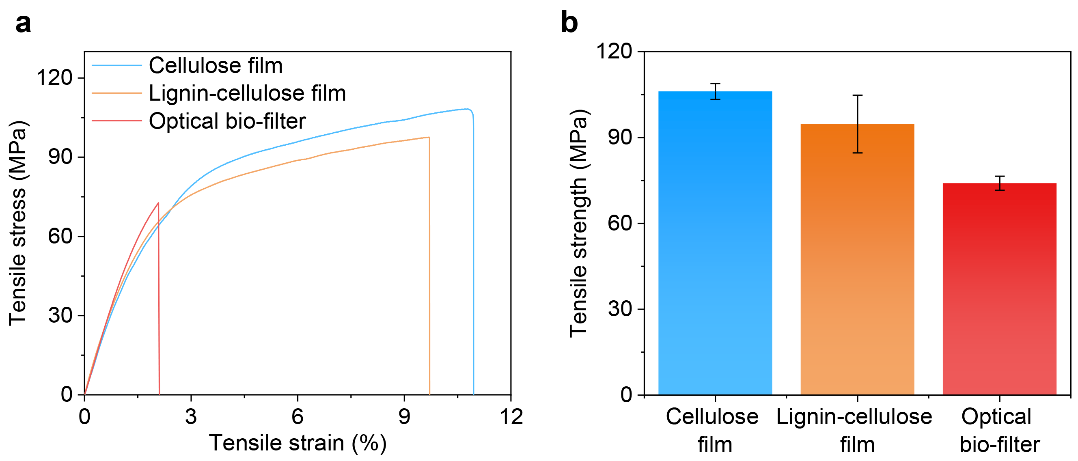


**Fig. S31.** Tensile strength of cellulose film, lignin-cellulose film, and optical bio-filter.


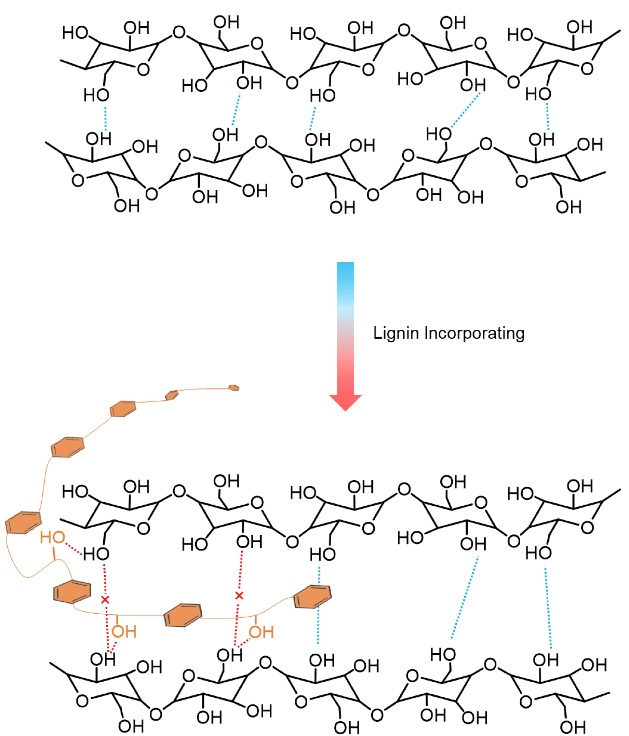


**Fig. S32.** Schematic of the effect of lignin on tensile strength of optical bio-filter.


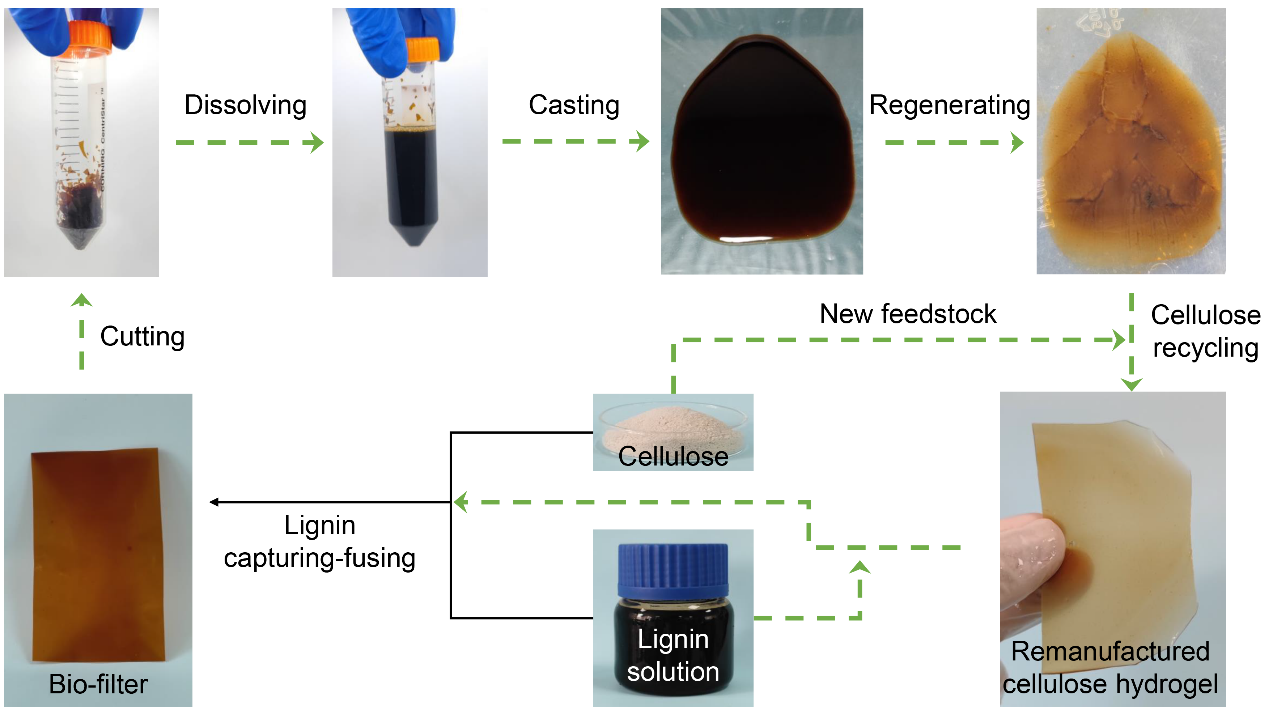


**Fig. S33.** Schematic of recyclability of the optical bio-filter.

Waste bio-filters were broken and then dissolved in aqueous BzMe_3_NOH solution. Considering that lignin can be deacetylated in an alkaline solution, we used NaOH (1 mol L^-1^) to remove lignin in hydrogels as much as possible and recycled cellulose by freeze-drying. Recycled cellulose (1.5 g) and new cellulose (0.6 g) were swelled in deionized water (7.5 g) and dissolved with 40 wt% aqueous BzMe_3_NOH (22.5 g) at 8 °C again, which was then processed into cellulose hydrogel. Finally, the remanufactured cellulose hydrogel could be used to obtain the bio-filter via our lignin capturing-fusing approach.


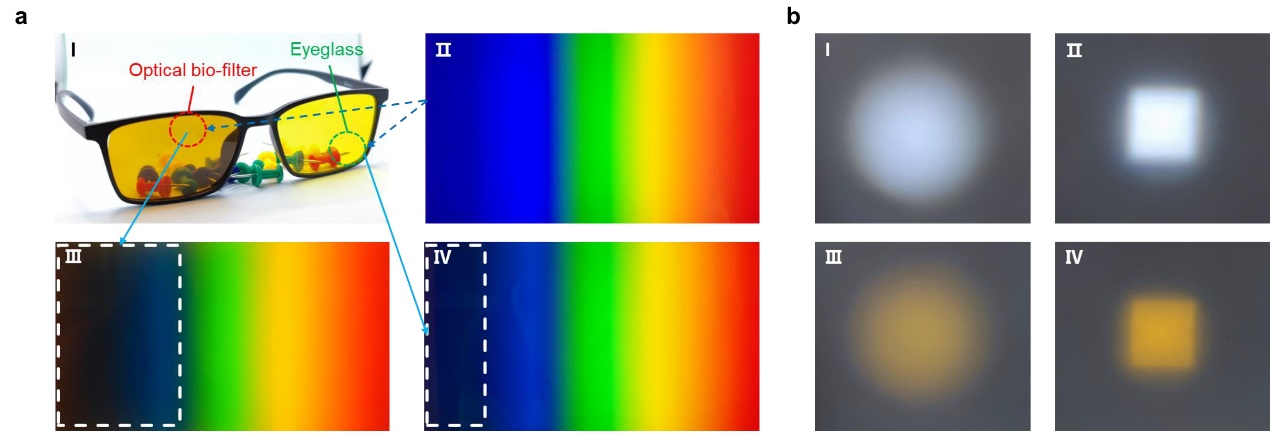


**Fig. S34.** (a) Application for reducing blue light with optical bio-filter. (b) Optical bio-filter does not change the shape of the light.

This application of the bio-filter in visible light shows reduction in the intensity of blue light. We fitted the filter to an eyeglass frame and found that it blocked blue light better than the anti-blue light eyeglasses that we purchased. Note that the bio-filter did not affect the shape of the propagated light.


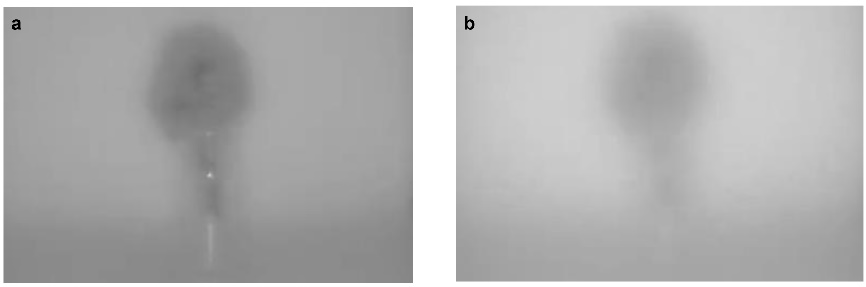


**Fig. S35.** Blur images of a monitor covered by cellulose film (a) and lignin-cellulose film (b).

# **Supplementary Notes**

**Note S1: Preparation of raw cellulose acetic acid pulping extracted from corncob**

The cellulose acetic acid pulping extracted from corncob was prepared according to our previous work [45]. Briefly, 100 g crushed and screened corncob was placed in a three-mouth flask. Acetic acid solution, 1 L of 95%, was injected, and the mixture was boiled and condensed by reflux for 1 h. The material was poured out after cooling, and an equal amount of 95% acetic acid solution accompanied by 1.5 mL concentrated sulfuric acid was added. The mix was further boiled and condensed for 2 h. Then, 3 g anhydrous sodium acetate was added as a neutralization reagent. Finally, the fine slurry separated by a pulp screen was stirred in 4 wt% NaOH solution for 24 h to obtain raw acetic acid lignocellulose after drying.

**Note S2: Preparation of corncob acetic acid lignin.**

Lignin solution produced from acetic acid pulping was poured into 6 times the volume of deionized water for sedimentation. The supernatant was poured off until the sugar was completely washed, and the remainder was frozen. The frozen solution was melted slowly at ambient temperature, and the melted water was removed. Finally, the lignin powder containing a small amount of water was freeze-dried to obtain corncob acetic acid lignin.

**Note S3: Fabrication of optical bio-filters with different lignin loading concentrations.**

Cellulose (1.8 g) extracted from corncob was swelled with deionized water (7.5 g) and dissolved using 40 wt% aqueous BzMe_3_NOH (22.5 g) at 8°C for 24 h. The cellulose solution was degassed by centrifuging at 6000 r/min for 5 min. Then, the corresponding hydrogel was obtained by regeneration in ethylene glycol (8°C). The regenerated cellulose hydrogel was washed with deionized water to remove chemicals, then immersed in 1/5/10 wt% aqueous lignin/acetic acid (90 vol%) for 5 h (hydrogel: lignin solution ≈ 1 g: 20 mL) to completely exchange the water in hydrogel. The hydrogel filled with lignin/acetic acid solution was immersed in deionized water to trap the lignin and obtain lignin-cellulose hydrogel_1%, lignin-cellulose hydrogel_5%, and lignin-cellulose hydrogel_10%. Subsequently, the lignin-cellulose hydrogel was dried at 97 °C using a Rapid-Köthensheet former (RK-3A, FrankPTI, Vorchdorf, Austria) to manufacture lignin-cellulose film_1%, lignin-cellulose film_5%, and lignin-cellulose film_10%. Finally, the optical bio-filter_1%, optical bio-filter_5%, and optical bio-filter_10% were manufactured by hot-pressing at 180 °C and 5 MPa for 1 h.

**Note S4: Preparation of bamboo and hardwood acetic acid lignin.**

Bamboo and hardwood acetic acid lignin were obtained from Yinnovator Biotech Co. Ltd. (Guangzhou, China). The lignin preparations were dissolved in 90 vol% aqueous acetic acid, forming 10 wt% lignin/ acetic acid solution. Then, lignin/ acetic acid solution was poured into 6 times the volume of deionized water for sedimentation. The supernatant was poured off until the sugar was completely washed, and the remainder was frozen. The frozen solution was melted slowly at ambient temperature, and the melted water was removed. Finally, the lignin powder containing a small amount of water was freeze-dried to obtain purified bamboo and hardwood lignins.

**Note S5: Cellulose from bamboo and hardwood.**

Bamboo dissolving pulp (DP = 500) was supplied by Sichuan Tianzhu Bamboo Resources Development Co., Ltd. (Sichuan, China).

Hardwood dissolving pulp (DP = 520) was supplied by Asia Symbol (Shandong) Pulp and Paper Co., Ltd. (Shandong, China).

**Note S6: Fabrication of optical bio-filter based on bamboo.**

Cellulose (1.5 g) extracted from bamboo was swelled with deionized water (9 g), and the swelled cellulose was dissolved with urea (3 g) and 40 wt% aqueous BzMe_3_NOH (18 g) at 8°C. The cellulose solution was degassed by centrifuging at 2000 r/min for 5 min after one freeze-thaw cycle. A hydrogel was obtained by regeneration in ethylene glycol (8°C). After being washed with deionized water, the hydrogel was immersed in 5 wt% lignin/acetic acid (90 vol%) for 5 h, (hydrogel: lignin solution ≈ 1 g: 20 mL) to completely exchange the water in the hydrogel. Then, the hydrogel filled with lignin/acetic acid solution was immersed in deionized water to trap the lignin and obtain lignin-cellulose hydrogel. Subsequently, the lignin-cellulose hydrogel was dried at 97 °C using a Rapid-Köthensheet former (RK-3A, FrankPTI, Vorchdorf, Austria) to manufacture lignin-cellulose film. Finally, the lignin-cellulose film was hot-pressed between two polyimide films at 180 °C and 5 MPa for 1 h to produce the optical bio-filter based on bamboo.

**Note S7: Fabrication of optical bio-filter based on hardwood.**

Cellulose (1.5 g) extracted from eucalyptus wood was swelled with deionized water (9 g), and the swelled cellulose was dissolved with urea (3 g) 40 wt% aqueous BzMe_3_NOH (18 g) at 8°C. The cellulose solution was degassed by centrifuging at 2000 r/min for 5 min after one freeze-thaw cycle. Then, a hydrogel was obtained by the regeneration in ethylene glycol (8°C). After being washed with deionized water, the hydrogel was immersed in 5 wt% lignin/acetic acid (90 vol%) solution for 5 h (hydrogel: lignin solution ≈ 1 g: 20 mL) to completely exchange the water in the hydrogel. Then, the hydrogel filled with lignin/acetic acid solution was immersed in deionized water to trap lignin to obtain lignin-cellulose hydrogel. Subsequently, the lignin-cellulose hydrogel was dried at 97 °C using a Rapid-Köthensheet former (RK-3A, FrankPTI, Vorchdorf, Austria) to manufacture lignin-cellulose film. Finally, the lignin-cellulose film was hot-pressed between two polyimide films at 180 °C and 5 MPa for 1 h to produce the optical bio-filter based on hardwood.

# **Supplementary Table**

**Table S1.** **Optical properties of materials based on biological materials.**

| Samples  (composition) | UV-shielding efficiency (%) | Transmittance  at 800 nm (%T) | Haze at 550 nm (%H) | Thickness (µm) | Ref. |
| --- | --- | --- | --- | --- | --- |
| DLNP  (lignocellulose) | 38.37 | 82.95 | 54.20 | 43±1 | [41] |
| HP-MFLC-K2  HP-MFLC-K65  HP-MFLC-K114  SE-MFLC-K2  SE-MFLC-K65  SE-MFLC-K114  (lignocellulose) | 33.74  88.83  93.69  78.29  95.59  97.75 | 88.22  70.63  54.32  51.17  48.02  32.16 | 71.98  22.25  76.67  21.53  78.29  78.30 | 40.7±1.8  40.2±1.6  45.6±1.9  51.8±0.4  52.0±2.0  51.6±0.5 | [43] |
| 21-LCNP  16-LCNP  11-LCNP  5-LCNP  (Lignin-containing cellulose) | 74.51  28.24  22.42  13.85 | 91.98  92.42  93.19  92.31 | 97.25  91.98  69.01  20.22 | 53  47  44  44 | [27] |
| Straw/WP 80/20  (Straw/wood pulp) | 90.07 | 58.48 | 79.45 | N/A | [46] |
| Cellulose film  (Cellulose) | 5.74 | 97.03 | 80.84 | 90 | [47] |
| NP-K2  NP-K65  NP-K114  MP-K2  MP-K65  MP-K114  (Lignocellulose) | 12.07  69.27  69.27  39.66  81.46  92.40 | 92.74  74.30  76.54  71.06  47.15  30.84 | 26.92  19.04  28.56  76.83  78.08  79.23 | 21.7±0.5  19.9±0.4  21.7±0.9  22.8±1.2  20.9±1.0  24.9±1.3 | [48] |

**Table S1. Continued.**

| Samples  (composition) | UV-shielding efficiency (%) | Transmittance  at 800 nm (%T) | Haze at 550 nm (%H) | Thickness (µm) | Ref. |
| --- | --- | --- | --- | --- | --- |
| TOCN film  (TEMPO-oxidized cellulose nanofibrils) | 16.98  19.99  20.42  21.04  22.71 | 88.33  85.52  84.80  84.06  82.92 | 3.54  32.19  47.81  60.31  61.04 | 29.6±5.1  32.6±2.3  39.6±1.8  41.0±2.5  42.5±1.9 | [28] |
| LNP  CNP  L-modified LNP  (Lignocellulose and cellulose) | 98.53  14.95  43.37 | 65.47  96.63  93.26 | 92.71  69.36  89.36 | ~45 | [49] |
| WS2  WS8  WS15  (Lignocellulose) | 94.08  95.23  95.78 | 8.87  6.86  6.09 | 88.7  90.1  89.6 | 42±3  43±4  47±10 | [45] |
| Transparent papers  (TEMPO-oxidized wood fibers and NFC) | 24.03  25.87  27.52  28.18  26.86 | 75.45  74.06  73.20  72.41  73.53 | 57.71  46.53  40.08  26.78  19.35 | 45–51 | [50] |
| Transparent wood  （Wood and polymers） | 26.74  25.97  17.38 | 85.16  89.66  89.35 | 76.42  81.07  69.88 | ~1500  ~1000  ~1000 | [51]  [40]  [39] |
| Cellulose film  Lignin-cellulose film  Optical bio-filter  (Lignocellulose) | 47.33  99.3  99.9 | 77.58  68.4  88.6 | 39.33  14.98  0 | 44.5±4.7  56.8±7.1  48.3±4.2 | This  work |
